# Computational evaluation of zirconocene catalysts for ε-caprolactone cationic ring-opening polymerization

**DOI:** 10.1038/s41598-024-54157-y

**Published:** 2024-02-17

**Authors:** Wijitra Meelua, Tanchanok Wanjai, Jitrayut Jitonnom

**Affiliations:** 1 Demonstration School, University of Phayao, Phayao, 56000 Thailand; 2 Unit of Excellence in Computational Molecular Science and Catalysis, and Division of Chemistry, School of Science, University of Phayao, Phayao, 56000 Thailand

**Keywords:** Catalyst design, Lactone, Zirconocene, Polymerization catalyst, Density functional theory, Chemistry, Theoretical chemistry, Electronic structure

## Abstract

This quantum chemical study presents the ligand effect and a structure–property relationship in the cationic ring-opening polymerization (CROP) of ε-caprolactone using zirconocene catalysts. We first examined the effects of catalyst structure on the initiation and chain propagation steps of the CROP process. A total of 54 catalyst structures were investigated to understand the influence of the ligand structure on the stability of the catalyst–monomer complex and polymerization activity. The properties of the catalysts were analyzed in terms of ancillary ligands, ligand substituents, and bridging units. Calculations showed that the polymerization follows a proposed cationic mechanism, with ring opening occurring via alkyl-bond cleavage. A correlation between complex stability and activation energy was also observed, with ligand substituents dominating in both steps. While the ancillary ligands directly affect the HOMO energy level, the bridges are mainly responsible for the catalyst geometries, resulting in reduced complex stability and higher activation energy for the propagation step. This study contributes to a better understanding of the structural characteristics of zirconocene catalysts, which offers guidance for improving CROP activities in lactone polymerization.

## Introduction

**Figure 1:**
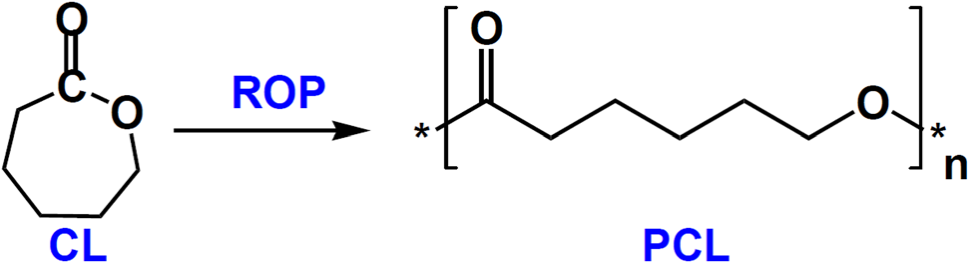
Ring-opening polymerization (ROP) of the cyclic monomer ɛ-caprolactone (CL).

Polycaprolactone (PCL) is a biodegradable, biocompatible and bioresorbable polymer that belongs to the family of aliphatic polyesters^1,2^. In past years, PCLs attract great attentions as well-known biomaterials with a plenty of applications in the fields of pharmacy, medicine, and biomedical engineering, including controlled-release drug delivery systems, absorbable surgical sutures, nerve guides, and three-dimensional (3-D) scaffolds^3–7^. PCLs are currently using as a promising target towards functionalized polymers^8,9^. PCLs are prepared by ring-opening polymerization of the cyclic monomer ɛ-caprolactone (CL) (Fig. 1), which can be obtained via cationic, anionic, or coordination mechanisms, depending on the catalytic system and the nature of active species^6,10,11^.

A wide range of catalysts/initiators have currently been used to promote the ring-opening polymerization of cyclic esters, including metal alkoxide complexes^12–14^, organometallic complexes^15–19^, and even enzymes^20^ (see a number of comprehensive reviews^6,10,11,21,22^ for more details). Among them, Group 4 metallocenes are gaining great attention in the synthesis of functionalized polymers via block-copolymerization^23–26^, besides their industrial use for olefin polymerization^21^. Because of the fact that Group 4 metallocene catalysts have long been used in olefin polymerizations, current research aims to expand their potential use for the production of the biomaterial PCL, using a rational design^27–29^ or a new synthesis route such as cationic ring-opening polymerization (CROP)^30^.

Zirconocene/borate catalytic systems^30^ have previously been used as effective catalysts for CROP of cyclic monomers (e.g., oxazolines^23,31^, lactones^24,25^, cyclic carbonates^26^) and linear monomers (e.g., vinyl ethers^32^ and methyl methacrylate^24^). The main limitation of these catalytic systems for polymer synthesis is their practical use, as controlling polymer molecular weight distribution during cationic polymerization is challenging^30^. Further studies to understand these catalytic processes at the atomic scale could help design and improve the polymerization catalyst of cyclic esters.

Tuning ligand framework on the catalyst structure with a combination of different catalytic systems^17,19,24,33^ has been reported as a strategy to improve the CROP activity with controlled molecular weight. The first cationic zirconocene complex system for the CROP of cyclic esters was reported by Hayakawa et al.^25,26^. The quantitative polymerization of CL in toluene at room temperature or 60 °C was obtained with afforded monodisperse PCLs (*M*_w_/*M*_n_ = 1.06–1.3). The best catalytic system was found for the methyl substituted ligand, (Me_5_Cp)CpZrMe_2_, as it polymerized CL with good control (*M*_w_/*M*_n_ < 1.13, *M*_n,calcd_ ≈ *M*_n,GPC_), and the molecular weight of the resulting PCLs increased with monomer conversion. They also suggested the higher catalyst efficiency as (Me_5_Cp)_2_ZrMe_2_ > (Me_5_Cp)CpZrMe_2_ > Cp_2_ZrMe_2_. Ten years later, a research group of Pitsikalis et al.^24^ reported the CROP of CL and δ-valerolactone using zirconocene catalytic systems with three different borate cocatalysts, B(C_6_F_5_)_3,_ [B(C_6_F_5_)_4_]^–^[Ph_3_C]^+^ and [B(C_6_F_5_)_4_]^–^[Me_2_NHPh]^+^. In this work, three zirconocenes were introduced in the reaction, namely the bis(cyclopentadiene) ligand (Cp_2_ZrMe_2_), bis(indene) ligand (Ind_2_ZrMe_2_), and tertiary butyl substituted ligand ((^t^BuCp)_2_ZrMe_2_) and the high molecular weights PCL and narrow molecular weight distributions (*M*_n_ = 10,000–38,000 g/mol and *M*_w_/*M*_n_ < 1.1) were obtained in a well-controlled manner. The catalyst performance was also reported as Cp_2_ZrMe_2_ < Ind_2_ZrMe_2_, and (^t^BuCp)_2_ZrMe_2_. The same research group also applied their catalytic systems for copolymerizations of other monomers such as methacrylate^24^, vinyl ether^32^ and 2-oxazoline^23,31^. The first mixed cyclopentadienyl-indenyl zirconocene, CpIndZrMe_2_, was used as a sole catalyst for the polymerizations of CL without adding any cocatalyst^33^. The resulting polymers yielded high quantitative outcome in terms of medium molecular weight polymers and moderate to broad polydispersities (*M*_n_ = 25,000–128,000 g/mol and *M*_w_/*M*_n_ = 1.11–1.98). These experimental studies open the way to enhance catalyst activities, polymer yields and molecular weights of the zirconocene polymerization by ligand modifications.

**Figure 2:**
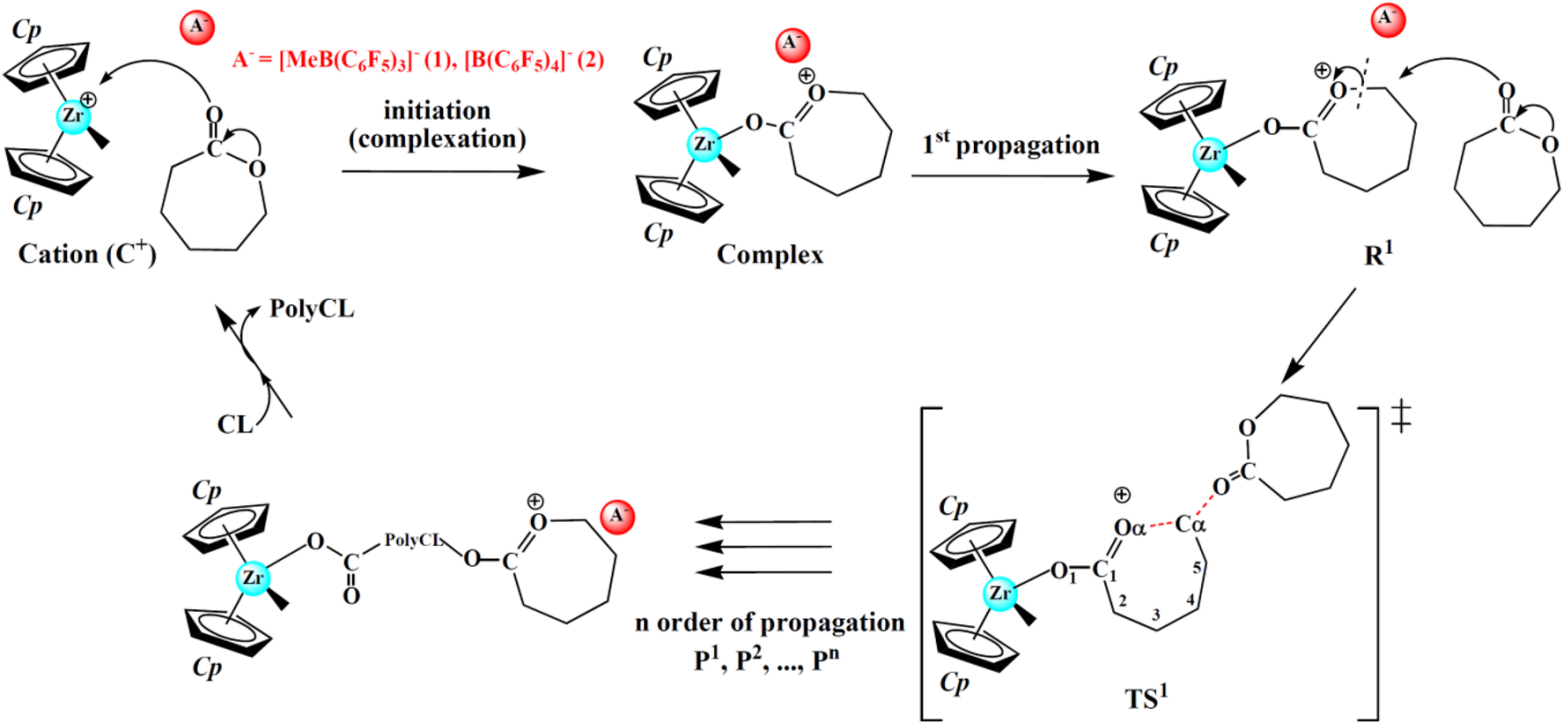
Mechanism of CROP of CL using zirconocene/borate systems. Important species involved in the initiation and propagation mechanism, including isolated Cp_2_ZrMe^+^ and CL, monomer-activated complex, reactant, transition state and product, are also shown and labeled as **Cation**, **Complex**, **R**^**n**^, **TS**^**n**^ and **P**^**n**^, respectively.

A number of density functional theory (DFT) studies on the metallocene-initiated CROP of cyclic esters and related monomers have also been reported (see references therein^34,35^), which provided valuable aspects of DFT modeling and visualization of cationic mechanisms and molecular structures in complement to experimental observations. For example, the electronic and geometric properties of dimethylzirconocene catalysts were greatly influenced by the presence of the silicon-bridge and ancillary ligands^19^. The effects of ligand structure, solvent, and metal on the dimerization of dinuclear zirconocene cations have also been investigated with DFT^36^. We also studied the influence of ligand structure on the initiation and propagation steps of the trimethylene carbonate polymerization using the naked model approach^17^. The CROP process follows an active chain end (ACE) mechanism where propagating species are tertiary oxonium ions located at the chain end (Fig. 2)^37,38^. In brief, a precatalyst Cp_2_ZrMe_2_ is activated by a borate cocatalyst (e.g., B(C_6_F_5_)_3_, [B(C_6_F_5_)_4_]^–^[Ph_3_C]^+^ and [B(C_6_F_5_)_4_]^–^[Me_2_NHPh]^+^), via a methide abstraction (known as catalyst activation) and yields the ion-pair [Cp_2_ZrMe]^+^[AMe]^–^ that is further separated into a cationic species Cp_2_ZrMe^+^ (**C**^**+**^) and a counteranion ([MeB(C_6_F_5_)_3_]^–^ (**1**) and [B(C_6_F_5_)_4_]^–^ (**2**)). The Cp_2_ZrMe^+^ is the active species in the polymerization process and that the propagation occurs through the ACE mechanism, where each propagating chain is initiated by a molecule of monomer (e.g. CL), corresponding to the initiation (or complexation, **Cation** → **Complex**) and propagation steps (**Complex** → **R1** → **P1**), depicted in Fig. 2.

Here, we employed the DFT modeling approach to study the effects of ligand structure through a series of zirconocene complexes (varying in steric hindrance and electronic properties) on the initiation and propagation. Their interactions with the boron cocatalysts on the rate-limiting step are analyzed in terms of the relative complex stability and the activation energies of the process. The structure–property relationship was then performed for the 54 zirconocene structures and the results were interpreted in terms of ancillary ligand, ligand substituents and bridge units. This study provides a better understanding of the structural characteristics of zirconocene catalysts in CL polymerizations.

## Computational methods

All geometries were optimized without restraints in gas-phase using density functional theory (DFT)^39,40^ at the B3LYP level. This B3LYP is chosen because of its success in DFT modeling studies on similar processes^7,13,17,35,41^ and its lower activation energy compared to other DFT functionals (Figure S1 of Supporting Information, SI). Zirconium atoms were treated with the effective core potential double-ζ basis set (LANL2DZ)^42^ whereas all non-metal atoms (C, H, O, B, and F) with a double-ζ basis set, 6–31G(d). The DFT/mixed basis set method has been largely applied for transition metal complexes^12,13,18,19,43^. Harmonic frequency calculations were performed to confirm the transition states and local minima obtained and to determine the Gibbs free energy (*G*) at standard temperature and pressure (298.15 K and 101.325 kPa). Accurate free energies and molecular electronic properties (Mulliken population analysis, HOMO–LUMO energies, and dipole moment) were obtained at the CPCM(toluene)-B3LYP-D3(BJ)/6–311++G(d,p) (LANL2DZ) level using single-point calculations on the optimized structures, incorporating implicit solvation and dispersion correction. All calculations were carried out using the Gaussian 09/16 software^29,44^.

## Results and discussion

**Figure 3:**
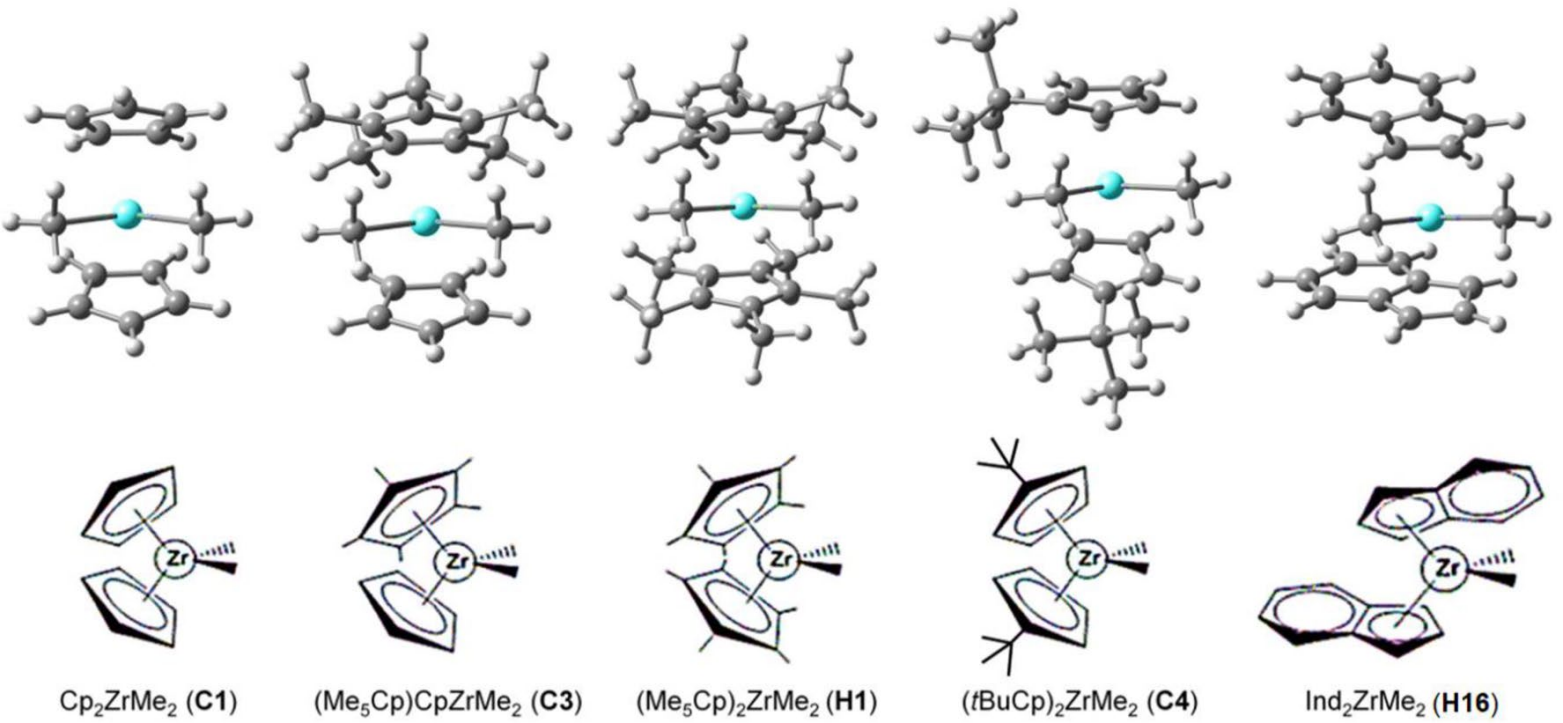
Five zirconocenes included for the study of catalyst structure effect. Nomenclatures used for each catalyst (**C1**, **C3**, **H1**, **C4** and **H16**) were assigned according to our recent DFT study on the zirconocene dimer properties^36^. The order of the dimer stability of these catalysts is **C1** > **C3** > **H16 >  > H1** > **C4**.

In this study, we evaluated the effects of zirconocene structures on the process by means of DFT calculations. Initially, we considered five catalysts that have been previously reported for their CROP activities^24,25^ and dimeric properties^36^, namely Cp_2_ZrMe_2_ (**C1**), (Me_5_Cp)CpZrMe_2_ (**C3**), (Me_5_Cp)_2_ZrMe_2_ (**H1**), (*t*BuCp)_2_ZrMe_2_ (**C4**) and Ind_2_ZrMe_2_ (**H16**) (Fig. 3). These catalysts were selected for comparison purpose, and their properties vary in steric hindrance (**C1**, **C3**, **H1**, and **C4**) and electronic properties (**C1** and **H16**). Their interactions with the two borate cocatalysts, [MeB(C_6_F_5_)_3_]^–^ and [B(C_6_F_5_)_4_]^–^, in the initiation and propagation were analyzed for the activity of the process. Detailed information on how to obtain the structures of zirconocene/borate systems can be found in SI.

### Catalyst structure influence on the process in the presence of cocatalyst

**Figure 4:**
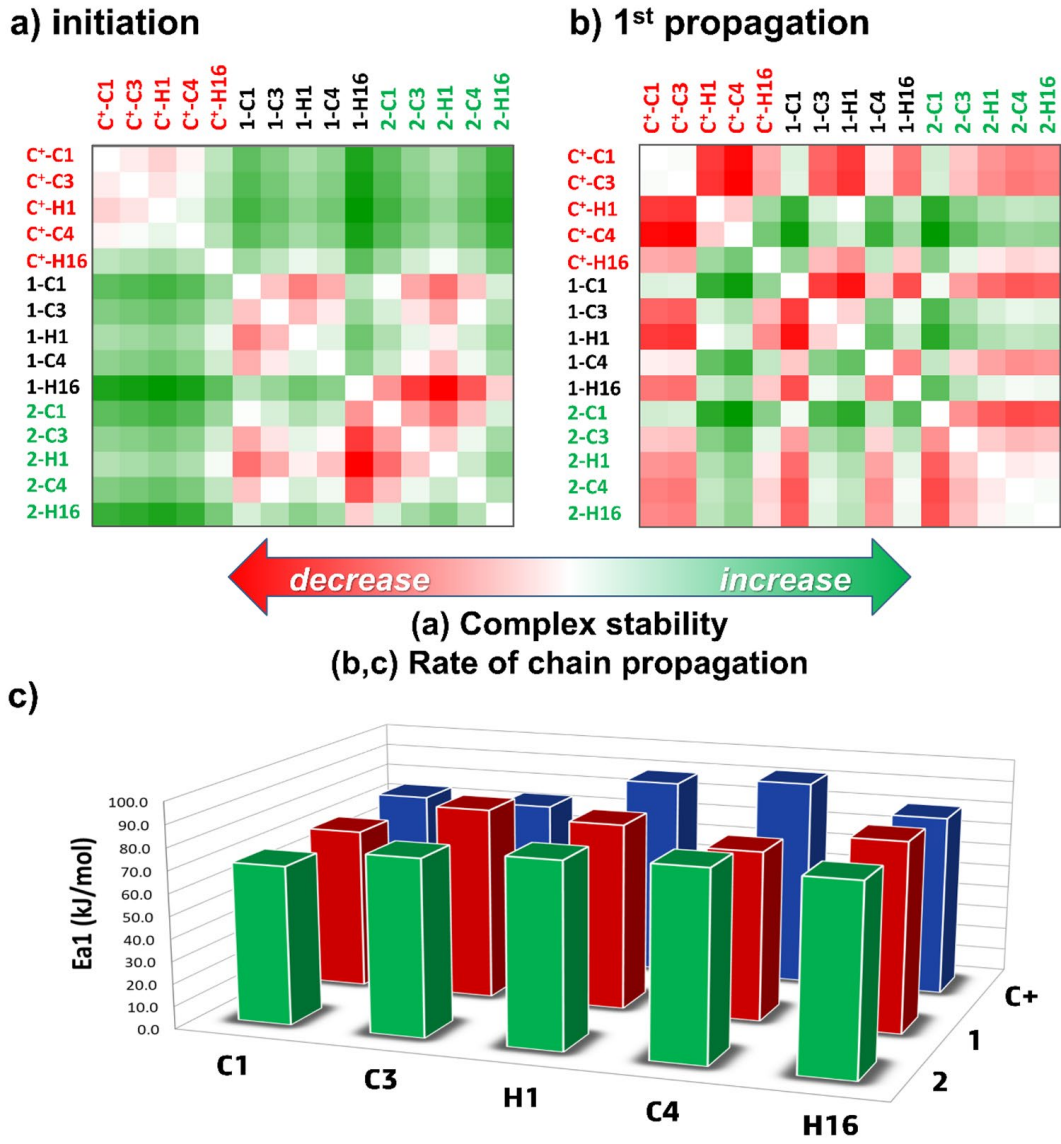
Heatmap corresponding to the energy cross-correlation between the catalyst pair for the (**a**) initiation and (**b**) first propagation. (**c**) Bar chart showing activation free energies (Δ*G*^ǂ^_Ea1_, kJ/mol) of the first propagation step using the selected catalysts (**C1**, **C3**, **H1**, **C4** and **H16**) for the three catalytic systems (**C**^**+**^, **1**, and **2**).

To evaluate the effects of zirconocene structures on the initiation and propagation using zirconocene/borate catalytic systems, five catalysts (**C1**, **C3**, **H1**, **C4**, and **H16**) that have been previously used in lactone polymerization^24,25^ were considered (see Fig. 3). Calculations of the initiation and propagation mechanisms were carried out using a similar procedure as previously reported in the literature^17^. Values of complexation energies for the **Cation → Complex** step (Δ*G*_com_) and activation Gibbs energies for the first and second propagations (Δ*G*^ǂ^_Ea1_ and Δ*G*^ǂ^_Ea2_) using the five zirconocenes of three different catalytic systems (**C**^**+**^, **1** and **2**) are also included in Table 1. The heatmap corresponding to the energy cross-correlation between the catalyst pair for the initiation, first and second propagations, as well as bar charts for the Δ*G*^ǂ^_Ea1_ values are depicted in Figs. 4 and S4. The ion-pair interactions between the different catalyst cations and the cocatalyst anions, [MeB(C_6_F_5_)_3_]^−^ and [B(C_6_F_5_)_4_]^−^, during the reaction were analyzed in terms of relative complex stabilities, activation free energies, and geometries, in comparison with the available experimental data.

**Table 1:** Values of complexation energies (Δ*G*_com_, **Cation → Complex**) and the activation energies in the first and second propagations (Δ*G*^ǂ^_Ea1_ and Δ*G*^ǂ^_Ea2_), as calculated for the initiation and the first and second chain propagation steps for zirconocene cation, X (**C**^**+**^), X/MeB(C_6_F_5_)_3_ˉ (**1**) and X/B(C_6_F_5_)_4_^–^ (**2**) systems (X = **C1**, **C3**, **H1**, **C4** and **H16**).

Systems	Initiation		Chain propagation				
	Δ*G*_com_	ΔΔ*G*	Δ*G*^ǂ^_Ea1_	ΔΔ*G*^ǂ^	Δ*G*^ǂ^_Ea2_	ΔΔ*G*^ǂ^	Ea2–Ea1
C^+^-C1	− 98.0	0.0	74.5	0.0	76.1	0.0	− 1.6
C^+^-C3	− 93.5	4.5	74.0	− 0.5	66.1	− 10.0	7.9
C^+^-H1	− 87.4	10.6	88.9	14.4	80.1	4.0	8.8
C^+^-C4	− 95.7	2.3	92.5	18.0	77.6	1.5	14.9
C^+^-H16	− 122.3	− 24.3	80.7	6.2	76.7	0.6	4.0
1-C1	− 158.5	0.0	71.6	0.0	71.3	0.0	0.3
1-C3	− 145.0	13.5	85.7	14.1	74.7	3.4	11.0
1-H1	− 129.5	29.0	88.9	17.3	76.5	5.2	12.4
1-C4	− 140.0	18.5	75.6	4.0	53.0	− 18.3	22.6
1-H16	− 183.6	− 25.1	84.5	12.9	67.2	− 4.1	17.3
2-C1	− 159.3	0.0	70.6	0.0	72.4	0.0	− 1.8
2-C3	− 138.7	20.6	78.6	8.0	80.1	7.7	− 1.5
2-H1	− 126.0	33.3	82.3	11.7	73.1	0.7	9.2
2-C4	− 145.0	14.3	83.8	13.2	74.2	1.8	9.6
2-H16	− 172.9	− 13.6	83.2	12.6	82.6	10.2	0.6

All energies are in units of kJ/mol, while those in parenthesis are the energy for each system relative to the energy of its counterpart C1.

### Relative complex stability and activation free energies

In the initiation step, a cationic complex is formed (**Cation** → **Complex**), which is hindered by the introduction of steric bulk, such as methyl and alkyl substituents, to the Cp ligands. This steric effect decreases the relative complex stability in the following order (Table 1; Fig. 4a): **C1** > **C4** > **C3** > **H1** for **C**^**+**^ (corresponding to the ∆*G*_com_ values of − 98.0, − 95.7, − 93.5, and − 87.4 kJ/mol, respectively), **C1** > **C3** > **C4 > H1** for **1** (− 158.5, − 145.0, − 140.0, and − 129.5 kJ/mol, respectively), and **C1 > C4** > **C3** > **H1** for **2** (− 159.3, − 145.0, − 138.7, and − 126.0 kJ/mol, respectively). However, changing the ancillary ligand from Cp (**C1**) to Ind (**H16**) greatly increases the complex stability, with ∆*G*_com_ values changing from − 98.0, − 158.5, and − 159.3 kJ/mol to − 122.3, − 183.6, and − 172.9 kJ/mol for **C**^**+**^, **1** and **2**, respectively. Note that these ligand effects have previously been shown as dominant in the zirconocene-initiated CROP of trimethylene carbonate^17^. The green color shown in the heatmap (Fig. 4a) further indicates that both cocatalyst anions strongly stabilize the cationic complex species of this step.

For the chain propagation, the activation free energies generally follow a trend **C**^**+**^  > **1** ~ **2** (Table 1 and Fig. 4b): the calculated barriers, Δ*G*^ǂ^_Ea1_, for **C1**, **C3**, **H1**, **C4**, and **H16** ranged from 74.0–92.5 kJ/mol for **C**^**+**^ to 71.6–88.9 and 70.6–83.2 kJ/mol for **1** and **2**, respectively. This effect is more profound for the second propagation (Δ*G*^ǂ^_Ea2_: **C**^**+**^  > **1** > **2**), which has lower barriers (ΔΔ*G* =  ~ 4 to 22 kJ/mol) compared to the first propagation (Δ*G*^ǂ^_Ea1_ > Δ*G*^ǂ^_Ea2_ and positive ΔΔ*G*, Table 1; Figure S4). The bar charts in Fig. 4c further show that the relative barrier heights, Δ*G*^ǂ^_Ea1_, follow the order **C1** < **C3** < **H1** for Cp_2_ ligand and **C1** < **H16** for Ind_2_ ligand, which account for the activity difference (e.g., *M*_n_, *M*_w_/*M*_n_; Table S1) reported by Hayakawa et al.^25^ and Pitsikalis et al.^24^. These results emphasize the influence of steric hindrance and bulkiness of the catalyst structures, which can be used as an important factor for ligand modifications.

### Geometric impact

**Figure 5:**
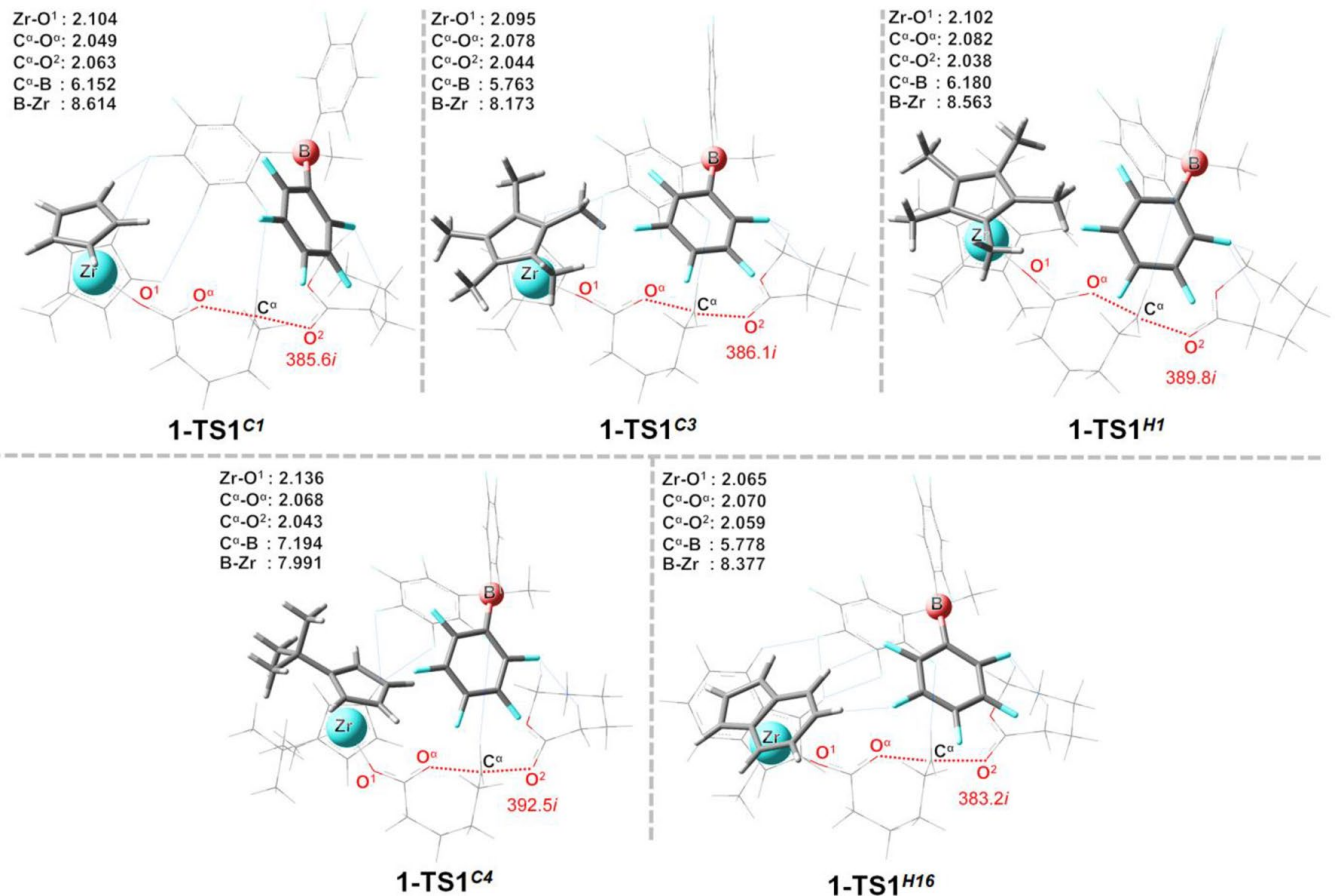
Optimized transition-state structures (**1-TS1**) in the first propagation step for different [X][MeB(C_6_F_5_)_3_]ˉ (**1**) systems (X = **C1**, **C3**, **H1**, **C4** and **H16**). Atoms in the top view are shown in the ball-and-stick model. All distances are in Å. Imaginary frequencies for each TS are also indicated. Atomic definitions discussed in the text are also given in the figure.

**Figure 6:**
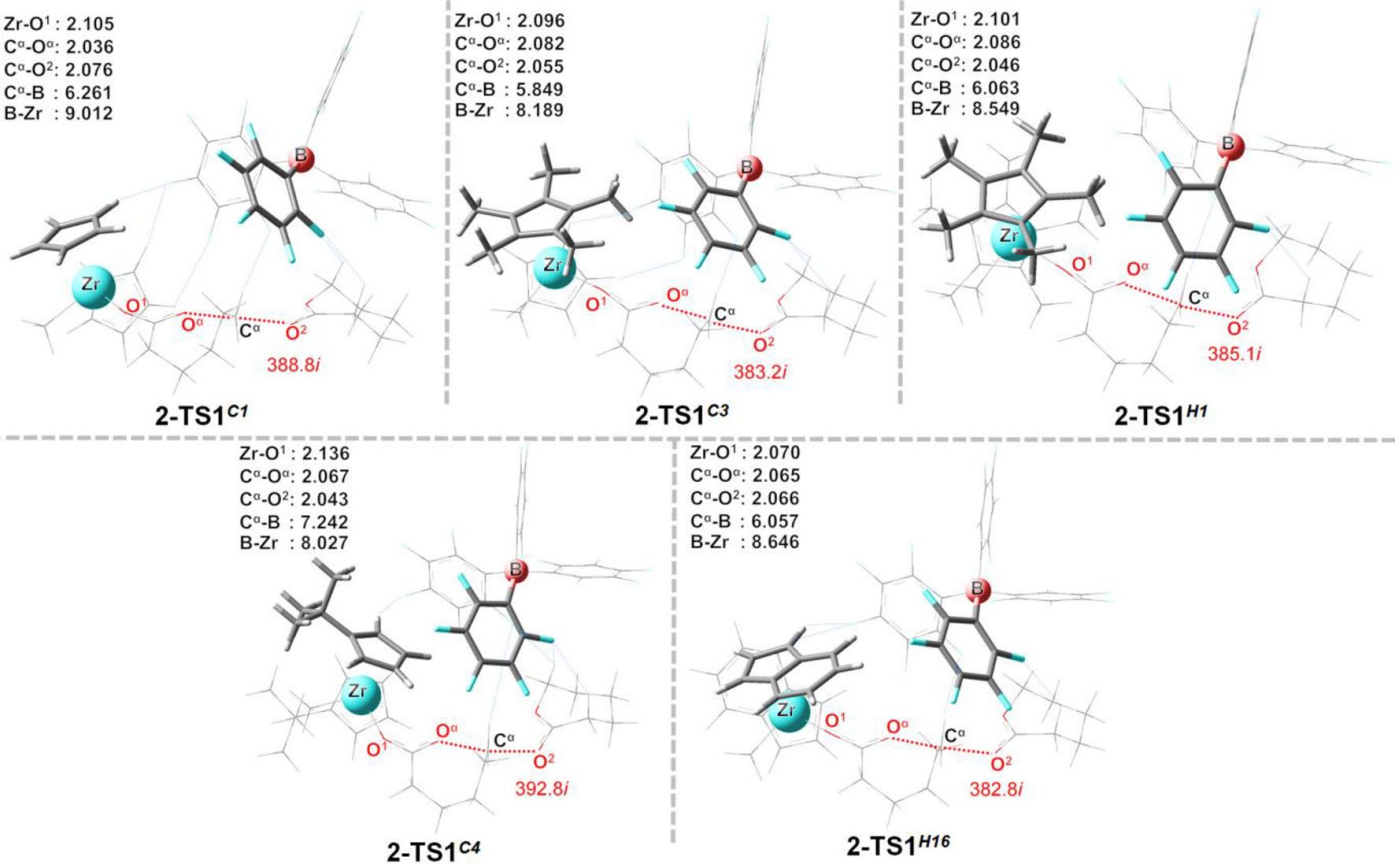
Optimized transition-state structures (**2-TS1**) in the first propagation step for different [X][B(C_6_F_5_)_4_]^–^ (**2**) systems (X = **C1**, **C3**, **H1**, **C4** and **H16**). Atoms in the top view are shown in the ball-and-stick model. All distances are in units of Å. Imaginary frequencies for each TS are also indicated. Atomic definitions discussed in the text are also given in the figure.

The optimized transition-state structures (**1-TS1** and **2-TS1**) in the first propagation step of [X][MeB(C_6_F_5_)_3_]ˉ (**1**) and [X][B(C_6_F_5_)_4_]^–^ (**2**) systems (X = **C1**, **C3**, **H1**, **C4**, and **H16**) are depicted in Figs. 5 and 6, respectively. Important geometric parameters for the first and second transition-state structures (**TS1** and **TS2**) for the three catalytic systems (**C**^**+**^, **1**, and **2**) are included in Table 2. As expected, the ligand structure affects mainly the geometries around the catalytic center of the propagating chain end, with small changes in the C^α^–O^α^ distance among the **C**^**+**^, **1**, and **2** systems. The presence of the counteranion results in the elongation of the C^α^–O^α^ bond by 0.02 Å, compared to that in the **C**^**+**^ system, and this bond becomes longer at **TS1**, with increasing of the methyl group on the Cp ligand (e.g., 2.049 Å at **1-TS1**^**C1**^, 2.078 Å at **1-TS1**^**C3**^, and 2.082 Å at **1-TS1**^**H**1^). Similar trend was also found in the system **2** (Fig. 6). This is in agreement with the observed difference in the catalyst activity^25^.

**Table 2:** Selected geometric parameters (Å) from the transition-state structures (**TS1** and **TS2**) for the naked cation (**C**^**+**^), [C^+^][MeB(C_6_F_5_)_3_]^–^ (**1**) and [C^+^][B(C_6_F_5_)_4_]^–^ (**2**) systems for different catalyst structures.

Catalyst	Distances	C^+^-TS1	C^+^-TS2	1-TS1	1-TS2	2-TS1	2-TS2
C1	Zr-O^1^	2.114	2.067	2.104	2.070	2.105	2.076
	Zr-B	–	–	8.614	8.096	9.012	7.862
	B-C^α^	–	–	6.152	5.292	6.261	5.736
C3	Zr-O^1^	2.120	2.094	2.095	2.094	2.097	2.091
	Zr-B	–	–	8.173	8.122	8.189	8.046
	B-C^α^	–	–	5.763	5.465	5.849	5.743
H1	Zr-O^1^	2.117	2.072	2.102	2.072	2.101	2.073
	Zr-B	–	–	8.563	9.517	8.549	9.524
	B-C^α^	–	–	6.180	5.304	6.063	5.718
C4	Zr-O^1^	2.113	2.069	2.136	2.072	2.136	2.078
	Zr-B	–	–	7.991	8.310	8.027	7.753
	B-C^α^	–	–	7.194	5.337	7.242	5.769
H16	Zr-O^1^	2.088	2.038	2.065	2.038	2.070	2.055
	Zr-B	–	–	8.377	8.972	8.646	8.255
	B-C^α^	–	–	5.778	5.113	6.057	6.867

The steric effect on the ligand structure is greater in **C4**, which has steric crowding on its Cp ligand. This limits a cocatalyst access near the C^α^ atom of the propagating chain end, as evidenced by the longest C^α^-B distance of 7.2 Å (see **1-TS1**^***C****4*^ in Fig. 5), compared to that in other systems.

There is a clear trend between the Zr-B distances and the steric degree on the Cp′ ligand, with Zr-B being significantly increased in the **TS2** for bulkier Cp′ ligands, i.e., **H1**, **C4**, and **H16**. For example, the catalyst **H1**, bearing a highly steric congestion on both Cp′ rings, yields the highest Zr-B distances of ~ 8.5 to 9.5 Å for both **1** and **2** (see Zr-B in Table 2). At **1-TS1**, Zr-B and B-C^α^ distances decrease in the order **C1** > **H1** > **H16** > **C3**. Overall, the results show that the size and shape of the Cp′ ligand could complicate the interactions between the boron cocatalyst and the catalyst.

### Structure–properties relationship

To find any factors that could account for the activation barriers of those five catalysts shown in Fig. 4c, we analyzed several electronic and geometric parameters, including HOMO–LUMO energies, atomic charge, and distances (M-Cen and C^α^–O^α^) involved in the catalytic center and the results are collected in Table 3. The Mulliken charges (*q*) of the metal center were analyzed at the **Complex** species and we found that the positive charge on the Zr atom correlates well with the observed activity difference of the Cp′ catalysts **(C1**, **C3**, and **H1**), i.e. the higher the charge on the Zr metal (*q*, **C1** < **C3** < **H1**), the higher the activation energies (Ea1, **C1** < **C3** < **H1**). Similar trends have been reported previously for styrene polymerization by a rare-earth-metal catalyst^45^. This is related to the donor–acceptor interaction between the ligand and the metal^46^ as can be seen from the longer distances between the Zr atom and the centroid of the Cp ring (Zr-Cen = 2.246, 2.274, and 2.289 Å in **2-C1**, **2-C3**, and **2-H1**, respectively, Table 3). There are significant trends between the energy gaps and the number of the methyl group on the Cp ring of the catalyst (Δ*E* values in Table 3) as well as those between the activation energies (Ea1) and the Zr-Cen distances, atomic charge, and Δ*E* values (Figure S5).

**Table 3:** Calculated Mulliken atomic charge (*q*, in unit of *e*) on Zr atom and HOMO and LUMO energies (*E*_HOMO_ and *E*_LUMO_, au)^a^ of **Complex** and energy gaps (Δ*E*, au)^a^ between the HOMO of CL and the LUMO of **Complex** for the naked cation (**C**^**+**^) and [X][A¯] systems (X = **C1**, **C3**, **H1**, **H16**, **C4**; A^−^  = [MeB(C_6_F_5_)_3_]^−^ (**1**) and [B(C_6_F_5_)_4_]^−^ (**2**)). Values of Zr-Cen and C^α^-O^α^ distances were also included.

Systems	Mulliken	Zr-Cen	C^α^–O^α^	*E*_HOMO_	*E*_LUMO_	Δ*E*_HOMO–LUMO_	Δ*E*^*b*^
**C**^**+***a*^	1.860	2.254	1.473	− 0.304	− 0.122	0.182	0.160
**2-C1**	1.788	2.246	1.495	− 0.243	− 0.095	0.148	0.187
**1-C1**	1.898	2.261	1.495	− 0.238	− 0.093	0.145	0.189
**2-C3**	1.915	2.261	1.490	− 0.244	− 0.084	0.160	0.198
**1-C3**	1.997	2.248	1.488	− 0.240	− 0.083	0.157	0.199
**2-H1**	2.242	2.289	1.488	− 0.236	− 0.086	0.150	0.196
**1-H1**	2.814	2.282	1.486	− 0.234	− 0.079	0.155	0.203
**2-H16**	1.907	2.271	1.494	− 0.238	− 0.101	0.137	0.181
**1-H16**	1.926	2.247	1.493	− 0.236	− 0.098	0.138	0.184
**2-C4**	1.969	2.255	1.492	− 0.242	− 0.090	0.152	0.192
**1-C4**	2.764	2.259	1.492	− 0.238	− 0.092	0.146	0.190

^a^Naked cation system (**C**^**+**^) for C1 catalyst as a minimum model for comparison.

^b^Δ*E* = *E*_LUMO_(active species, **Complex**) −*E*_HOMO_(CL). *E*_HOMO_(CL) =  − 0.28181 au.

**Figure 7:**
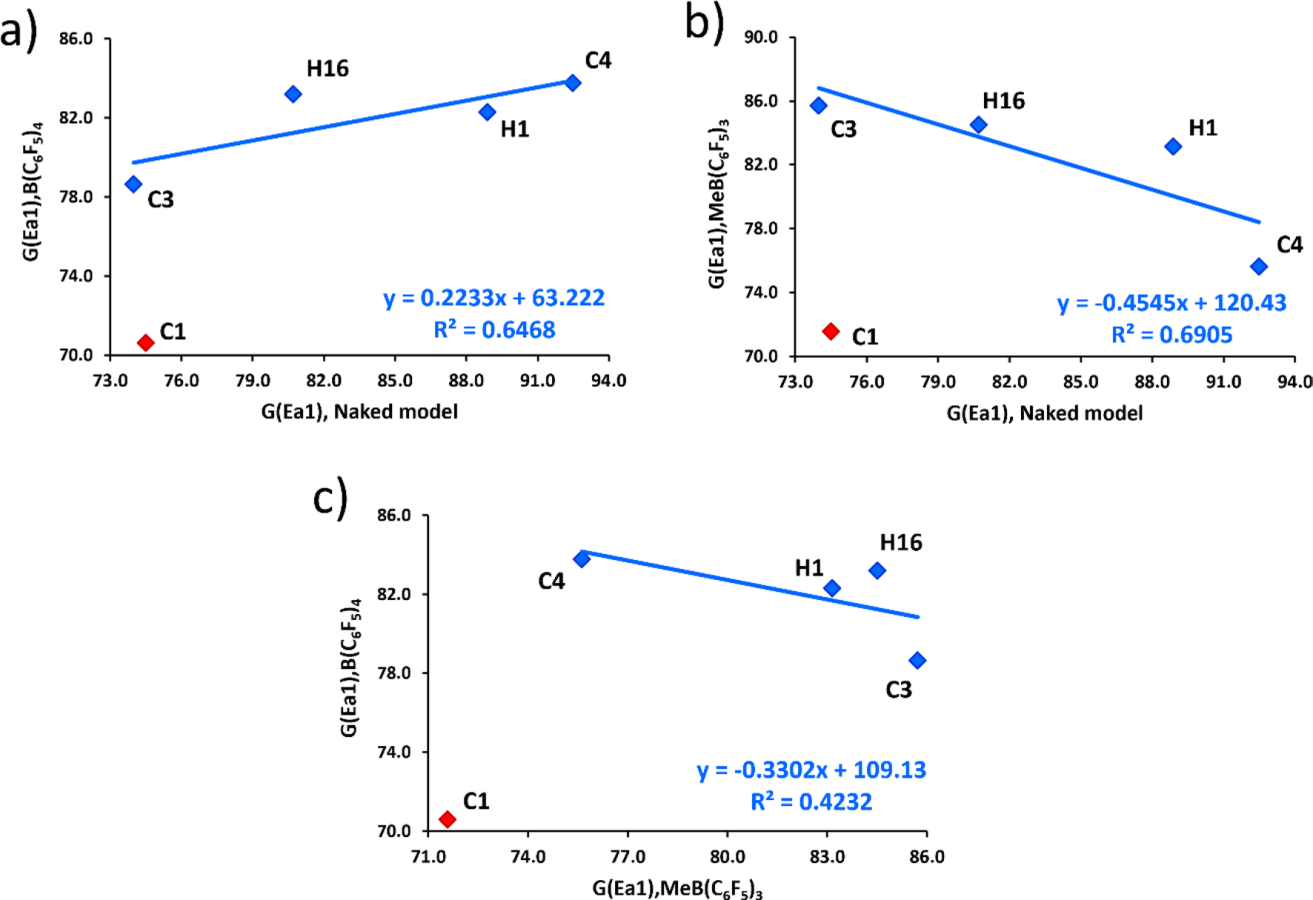
Relationship between the two activation Gibbs energies (Δ*G*^ǂ^_Ea1_) obtained from different catalytic model systems: (**a**) naked model vs B(C_6_F_5_)_4_; (**b**) naked model vs MeB(C_6_F_5_)_3_ and (**c**) MeB(C_6_F_5_)_3_ vs B(C_6_F_5_)_4._ Values are in units of kJ/mol. C1 is an outliner, which does not include in the equation.

To evaluate further whether the naked cation model could be a representative of the metallocene/borate system, we compared the two activation Gibbs energies of the rate-limiting step among the three catalytic systems (Fig. 7). We observed significant correlations between the activation energies, Ea1, from the naked model and the full models of [B(C_6_F_5_)_4_]^–^ and [MeB(C_6_F_5_)_3_]^–^ containing systems, with R^2^ of 0.6468 and 0.6905, respectively. These relationship analyses further suggest us that it is possible to use the naked cation approach as a pre-screening strategy for catalyst design for CROP of cyclic ester.

### Catalyst design with the naked model

**Figure 8:**
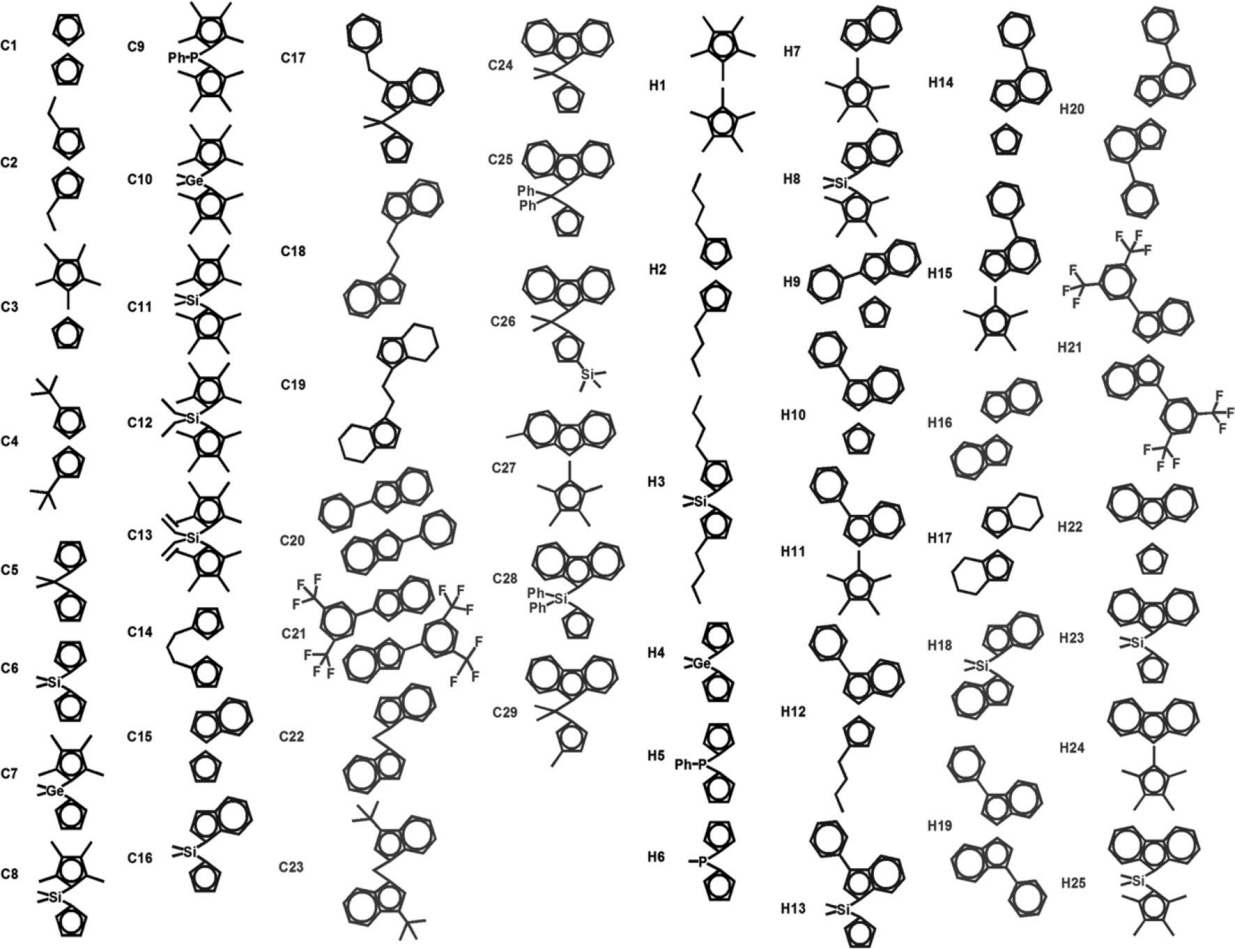
Schematic ligand structures of the studied zirconocenes.

**Figure 9:**
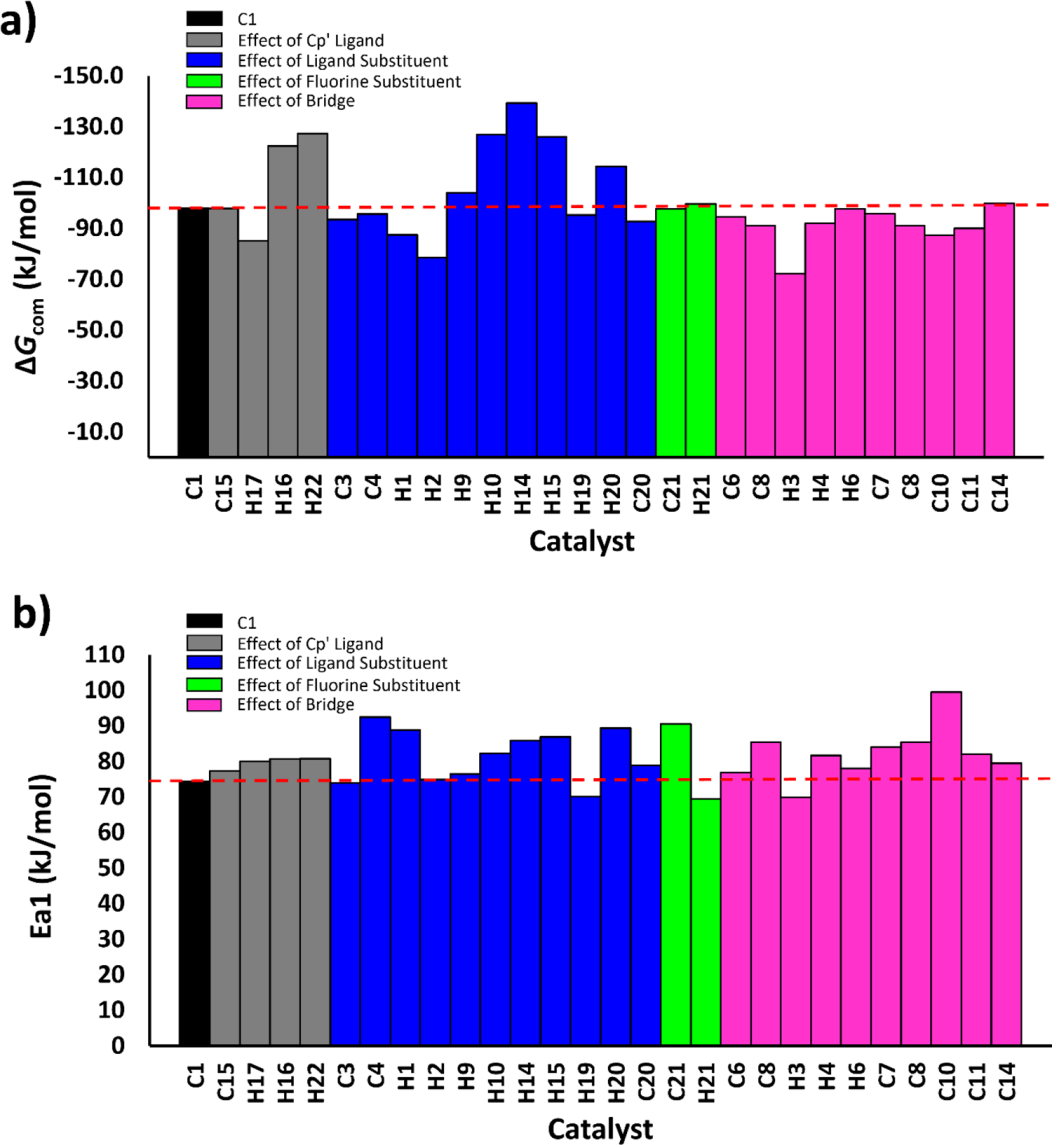
Bar chart showing (**a**) complexation energies, ∆*G*_com_, and (**b**) activation free energies of the first propagation (Ea1) for different catalyst ligands. The energies of **C1** (∆*G*_com_ = − 98.0 kJ/mol and Ea1 = 74.5 kJ/mol) are indicated with dashed lines.

Previous structure–activity relationship studies have proven that structural and electronic properties of metallocene catalyst can be tunable by quantum chemistry calculations for CROP^17–19,36^ and olefin polymerization^27,28,47,48^. As the results shown above for a small set of zirconocenes, the naked cation approach could be used as a simple strategy in pre-screening a large data set of compounds for candidate catalysts (Fig. 8). To expand the data set, we employed a larger data set of 54 zirconocene structures and used them for in silico pre-screening calculations with the naked model. We also used the same energetic parameters previously described for the CROP activity, i.e., Δ*G*_com_ for the relative complex stability (**Cation** → **Complex**) and Ea1 for the activation energy in the first propagation step (**R1** → **TS1**). From this data set, 29 optimized structures were taken from our previous study^16^ and additional 25 structures were built and optimized, leading to a total of 54 catalyst structures. Nomenclature of each catalyst structures (C_n_ and H_n_) was assigned in a similar fashion as previously reported by Karttunen et al.^48^ where C_n_ and H_n_ represent the available crystallographic structures and in silico design, respectively. The resulting structures and energetics as well as electronic properties for the 54 zirconocenes are collected in Tables S2 and S3. Bar charts showing the ∆*G*_com_ and Ea1 values are plotted in Fig. 9. Using the **C1** energies as a reference, it is clear that both ancillary Cp′ ligand and ligand/fluorine substitutions significantly alter the ∆*G*_com_ and Ea1 values, and the latter has a strong effect. Other structural and electronic characteristics of zirconocene catalysts in CL polymerizations were described as ancillary ligand, ligand substituent and bridge below.

#### Effect of ancillary Cp′ ligand

The effect of Cp′ ligands on electronic and steric properties of the complexes can be seen by considering the three Cp′ ligands (Cp, Ind, Flu). Given the data in Tables S2 and S3 and Fig. 9, it is seen that changing Cp into more electron-rich ligand Ind or Flu clearly affects both electronic and steric nature of the catalyst in the process. This type of modification has a greater effect on the complex formation (up to 29 kJ/mol) as it increases the complex stability with a trend **C1** ≈ **C15** < **H16** < **H22** (corresponding to the ∆*G* values of − 98.0, − 97.9, − 122.3 and − 127.3 kJ/mol, respectively, Table S2). However, this effect yields a slight increase in the Ea1 values in the order: **C1** (74.5 kJ/mol) < **C15** (77.4 kJ/mol) < **H16** (80.7 kJ/mol) < **H22** (80.8 kJ/mol). These results are in-line with our previous study^17^ and support the slower kinetics observed for larger ancillary ligands^24^.

The changes in electronic properties due to the larger ancillary ligands can be seen from the atomic charge and HOMO–LUMO energies. The positive charges of Zr atoms become higher as the reaction proceeds from **Cation** to **Complex** (Cation/Complex): **C1** (1.537*e*^*−*^/1.861*e*^*−*^), **C15** (1.807*e*^*−*^/1.893*e*^*−*^), **H16** (1.672*e*^−^/2.007*e*^*−*^), and **H22** (1.647*e*^*−*^/1.886*e*^*−*^). The HOMO energies are strongly perturbed, with the **Cation** species being most affected (Figure S6a). This ancillary effect also shows a trend with ∆*E*_HOMO-LUMO_ values at **Complex** and **R1** (Figure S6b). The electronic origin of this modification is evident in the complexation energies of **H16** and **H17** (*−* 122.3 vs *−* 85.1 kJ/mol, respectively). The loss of a benzene ring in **H16** into a cyclohexane in **H17** creates a steric clash between the cyclohexane ring and the methylene carbon of the CL monomer (Figure S7). However, this effect is insignificant to the activation barrier of the first propagation (80.7 vs 79.9 kJ/mol for **H16** and **H17**).

#### Effect of ligand substituent

The substituent effects were analyzed by considering three different ligands (Cp_2_, Ind_2_, or CpInd) substituted by methyl, alkyl, or phenyl groups. The methyl substituents significantly decreased the relative stability of the complex, with **C1** (*−* 98.0 kJ/mol) compared to **C3** (*−* 93.5 kJ/mol) and **H1** (*−* 87.4 kJ/mol). The effect also raises the activation free energies up to ~ 15 kJ/mol when comparing **C3** to **H1** and **C7** to **C10** (Fig. 9b; Table S3). This can be explained by the electron-donating ability of the methyl substituent. The alkyl substituents also had the same effect, i.e., destabilized the complex and increased the activation energies. The effects of the alkyl group, however, are more complicated than those of methyl groups, as steric crowding might limit monomer access depending on their location on the Cp′ ring. This can be seen in **C2**, **H2**, and **C4** (Figures S8, S9). For example, **C2** has four possible conformations (namely, C2[1,2′], C2[1,3′], C2[1,4′], and C2[1,5′]), and their relative stabilities slightly differ upon steric repulsion with respect to the monomer (Figures S10–S13). The relative stability of the complex follows the trend C2[1,3′] < C2[1,2′] < C2[1,5′] < C2[1,4′] (Figure S10), while this trend is C2[1,5′] < C2[1,3′] < C2[1,4′] < C2[1,2′] for activation energies of the first propagation (Figure S13). A longer linear alkyl chain leads to a more stable complex, while the effect becomes stronger for the branch alkyl group (**C2**, **H2** vs **C4**, Figure S8). The complexation and activation energies are higher, up to ~ 25 and ~ 20 kJ/mol (**C4** vs **H2**, Figs. 9 and Figs. S8, S9), respectively. Overall, the alkyl substituents containing longer or steric ligands would lower the stability of the complex and raise the activation energies of the propagation.

The phenyl substituent destabilizes the complex and increases the activation energy, which varies depending on the position of the phenyl group. It is expected that the electron-donating ability of the phenyl group would make the cation less reactive towards the coordination of the monomer. This effect is observed in **H16**, **C20**, **H19**, and **H20** (Figures S14, S15), with relative complex stability decreasing as 4-phenyl (**H20**; − 114.4 kJ/mol) > 3-phenyl (**H19**; − 95.3 kJ/mol) > 2-phenyl (**C20**; − 92.8 kJ/mol), and activation energies increasing as 3-phenyl (**H19**; 70.1 kJ/mol) < 2-phenyl (**C20**; 78.9 kJ/mol) < 4-phenyl (**H20**; 89.4 kJ/mol). Similar effects can be found for the CpInd ligand (see **H9**, **H10**, and **H14** in Figures S16, S17): the complex stability decreases as 4-phenyl (**H14**) > 3-phenyl (**H10**) > 2-phenyl (**H9**), whereas activation energy increases as 2-phenyl (**H9**) < 3-phenyl (**H10**) < 4-phenyl (**H14**). When electron-withdrawing fluorine (F) is introduced into the phenyl substituent, it significantly affects the activation energies when comparing **C20** and **C21**: their relative energies of ∆*G*_com_ and Ea1 differ by ~ 5 and ~ 12 kJ/mol, respectively. The data in Fig. 9b also indicates that **H19** and **H21**, bearing 3-phenyl (indene) and 3-(3,5-di(trifluoromethyl)phenyl)indene ligands, exhibited the lower Ea1 values (70.1 and 69.4 kJ/mol) compared to that for **C1**.

#### Effect of bridge

**Figure 10:**
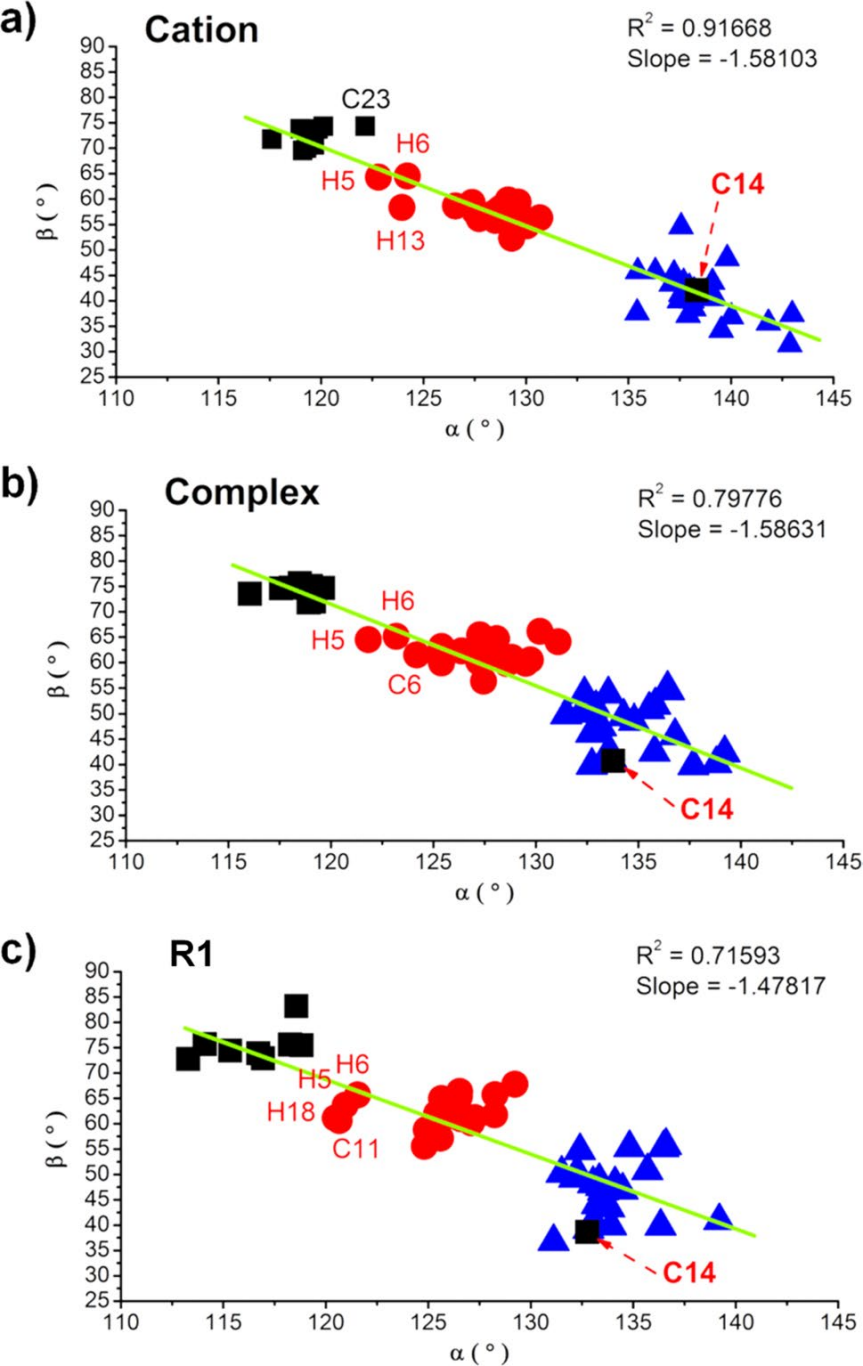
Correlation between the Cen-M-Cen (α) and Cen–Cen (β) angles in all catalyst structures at different intermediate species: (**a**) **Cation**, (**b**) **Complex**, and (**c**) **R1**. Three clusters **I–III** of zirconocenes can be classified, according to the α/β angles, and are shown in black, red and blue, respectively.

The effect of bridge can be seen by comparing **C1** to **C5**, **C6**, **H4**, **H6** and **C14**, **C3** to **C7** and **C8**, **H1** to **C10**, **C11**, **C12**, and **C13**, **H16** to **H18**, **C22**, and **C18**, **H22** to **C24** and **H23**, and others (**H2** vs **H3**, **C15** vs **C16**, **H7** vs **H8**, **H10** vs **H13**, and **H24** vs **H25**). As in other ligand effects, the bridge ligands generally destabilize all intermediates, leading to the lower stabilization to the complex and the higher activation energies to the first propagation. Longer bridge ligands also show similar effects, as can be seen in **C1**, **C5**, and **C14** for Cp_2_ ligand or **H16**, **C22**, **C18** for Ind_2_ ligand (Figure S18). This can be explained from the structural constraint found along the reaction intermediates, which can be justified from the Cen–Zr–Cen (α) and Cen–Cen (β) angles where Cen is the centroid of the Cp′ ring. We analyzed these angles for all 54 catalysts and found three different clusters (Fig. 10): carbon bridge ligands (8 catalysts; **cluster I**), non-carbon bridge ligand (Si, Ge, P; 21 catalysts; **cluster II**) and unbridged ligands (25 catalysts; **cluster III**). All these data can be found in Tables S2 and S3. The **cluster I** show more open structures of catalyst, which was characterized by the highest β values of around 69°–75°. This can be rationalized from the smallest atomic radius of bridging carbon compared with the other atoms (Si, Ge, P). **Cluster II** also shows similar characteristics of the bridged ligand but to a lesser extent (β ~ 55° to 65° and α ~ 122° to 132°). The **cluster III** is the largest members representing the unbridged ligands, with a narrow Cp–Cp structure (β ~ 34° to 55° and α ~ 131° to 142°). Excellent linear correlation (R^2^ = 0.91668) is found when plotting between the α and β angles at **Cation** and the values are significantly reduced when going to **Complex** and **R1** (Fig. 10).

Furthermore, bridge ligands can be used for selective coordination of monomers, particularly for CpInd ligands. They can be used for selective insertion from either a five-membered or a six-membered ring. Comparing **C15** (unbridged) with **C16** (bridged), the unbridged case shows similar energetics for both insertions **C15**(5) and **C15**(6) (− 98.0 vs − 97.9 kJ/mol for ∆*G*_com_ and 76.1 vs 77.4 kJ/mol for Ea1, respectively). However, due to the bridge, complex formations with the five-membered insertion of **C16**(5) are 26 kJ/mol more stable than those with the six-membered insertion of **C16**(6), while the activation energy remains unchanged (76.0 vs 74.1 kJ/mol for **C16**(5) and **C16**(6), Figure S19).

Despite the destabilizing effect by the bridge, there is an exception when the Cp′ ligand substituted by the electron-donating group. This can be seen in **H3**, which possess butyl substituent on the Cp ring and a silicon bridged atom, is found to exhibit the lower activation energy (69.9 kJ/mol) compared to that of **C1** (Fig. 9b). Overall, the results indicate that the catalytic performance relies not only on the catalyst structure but also on the electronic nature of the ligand, which has a profound effect on the reaction performance.

## Conclusion

We present a structure–property relationship in the CL CROP reaction on the 54 zirconocene structures using DFT calculations. Catalyst structure effect is then investigated as an important factor for tuning the CROP activity, which is analyzed in terms of ancillary ligands, ligand substituents, and bridging units. The polymerization was confirmed to proceed via a cationic mechanism where the ring opening taking place via alkyl-bond cleavage. The activation free energies calculated for the insertion of the first monomer into the C^α^–O^α^ bond within the CL ring for **C1**, **C3**, and **H1** does correlate with the observed difference in the catalyst activity reported experimentally. The calculations also show that ligand substituent has a dominant effect to the reaction, especially the electron-donating groups of alkyl and phenyl substituents. The ancillary ligands showed a direct effect on electronic energies, while the bridges are mainly responsible for the catalyst geometry leading to a decrease in the complex stability and an increase in the activation energy of the propagating step. From the structure–property analysis, we demonstrate that the naked model approach can be applied to prescreen catalyst candidate for the CROP process. Overall, this study contributes to a better understanding of the structural characteristics of metallocene catalysts in CL polymerizations, paving the way for further investigations and advancements in this field.

### Supplementary Information

Supplementary Information 1.

Supplementary Information 2.

## Data Availability

The data that support the findings of this study are available in the supplementary material of this article.

## References

[1] Goossens H (2013). Cationic ring-opening polymerization of 2-propyl-2-oxazolines: Understanding structural effects on polymerization behavior based on molecular modeling. ACS Macro Lett..

[2] Mirmohammadi SA, Imani M, Uyama H, Atai M (2014). Hybrid organic-inorganic nanocomposites based on poly(ϵ-caprolactone)/polyhedral oligomeric silsesquioxane: Synthesis and in vitro evaluations. Int. J. Polym. Mater. Polym. Biomater..

[3] Schmitt PR, Dwyer KD, Coulombe KLK (2022). Current applications of polycaprolactone as a scaffold material for heart regeneration. ACS Appl. Bio Mater..

[4] Espinoza S (2020). Poly-ε-caprolactone (PCL), a promising polymer for pharmaceutical and biomedical applications: Focus on nanomedicine in cancer. Int. J. Polym. Mater. Polym. Biomater..

[5] Abudula T (2020). Sustainable drug release from polycaprolactone coated chitin-lignin gel fibrous scaffolds. Sci. Rep..

[6] Jérôme C, Lecomte P (2008). Recent advances in the synthesis of aliphatic polyesters by ring-opening polymerization. Adv. Drug Deliv. Rev..

[7] Kaluzynski K (2022). Cationic polymerization of cyclic trimethylene carbonate induced with initiator and catalyst in one molecule: Polymer structure, kinetics and DFT. J. Catal..

[8] Kaluzynski K, Pretula J, Lewinski P, Kaźmierski S, Penczek S (2022). Synthesis and properties of functionalized poly(ε-caprolactone); chain polymerization followed by polycondensation in one pot with initiator and catalyst in one molecule. synthesis and molecular structures. Macromolecules.

[9] Mirmohammadi SA (2014). The effects of solvent and initiator on anionic ring opening polymerization of ϵ-caprolactone: Synthesis and characterization. Polym. Int..

[10] Nuyken O, Pask SD (2013). Ring-opening polymerization—an introductory review. Polymers (Basel).

[11] Mirmohammadi SA, Imani M, Uyama H, Atai M (2013). In situ photocrosslinkable nanohybrids based on poly(ε-caprolactone fumarate)/polyhedral oligomeric silsesquioxane: Synthesis and characterization. J. Polym. Res..

[12] Jitonnom J, Meelua W (2020). DFT study of lactide ring-opening polymerizations by aluminium trialkoxides: Understanding the effects of monomer, alkoxide substituent, solvent and metal. Chem. Phys. Lett..

[13] Jitonnom J, Molloy R, Punyodom W, Meelua W (2016). Theoretical studies on aluminum trialkoxide-initiated lactone ring-opening polymerizations: Roles of alkoxide substituent and monomer ring structure. Comp. Theor. Chem..

[14] Sattayanon C (2014). Theoretical study on the mechanism and kinetics of ring-opening polymerization of cyclic esters initiated by tin(II) n-butoxide. Comp. Theor. Chem..

[15] Kaluzynski K, Pretula J, Lewinski P, Kaźmierski S, Penczek S (2020). Catalysis in polymerization of cyclic esters Catalyst and initiator in one molecule. Polymerization of ε-caprolactone. J. Catal..

[16] Jitonnom J, Meelua W (2018). Data on electronic structures for the study of ligand effects on the zirconocene-mediated trimethylene carbonate polymerization. Data Brief.

[17] Jitonnom J, Meelua W (2017). Effect of ligand structure in the trimethylene carbonate polymerization by cationic zirconocene catalysts: A “naked model” DFT study. J. Organomet. Chem..

[18] Jitonnom J, Meelua W (2017). Cationic ring-opening polymerization of cyclic carbonates and lactones by group 4 metallocenes: A theoretical study on mechanism and ring-strain effects. J. Theor. Comput. Chem..

[19] Jitonnom J, Meelua W (2014). Effects of silicon-bridge and π-ligands on the electronic structures and related properties of dimethyl zirconocene polymerization catalysts: A comparative theoretical study. Chiang Mai J. Sci..

[20] Elsässer B, Schoenen I, Fels G (2013). Comparative theoretical study of the ring-opening polymerization of caprolactam vs caprolactone using QM/MM methods. ACS Catal..

[21] Collins RA, Russell AF, Mountford P (2015). Group 4 metal complexes for homogeneous olefin polymerisation: A short tutorial review. Appl. Petrochem. Res..

[22] Ajellal N (2010). Metal-catalyzed immortal ring-opening polymerization of lactones, lactides and cyclic carbonates. Dalton Trans..

[23] Kourti M-E, Fega E, Pitsikalis M (2016). Block copolymers based on 2-methyl- and 2-phenyl-oxazoline by metallocene-mediated cationic ring-opening polymerization: Synthesis and characterization. Polym. Chem..

[24] Kostakis K, Mourmouris S, Karanikolopoulos G, Pitsikalis M, Hadjichristidis N (2007). Ring-opening polymerization of lactones using zirconocene catalytic systems: Block copolymerization with methyl methacrylate. J. Polym. Sci. A Polym. Chem..

[25] Hayakawa M, Mitani M, Yamada T, Mukaiyama T (1997). Living ring-opening polymerization of lactones using cationic zirconocene complex catalysts. Macromol. Chem. Phys..

[26] Hayakawa M, Mitani M, Yamada T, Mukaiyama T (1996). Living ring-opening polymerization of cyclic carbonate using cationic zirconocene complex as catalyst. Macromol. Rapid Commun..

[27] Laine A (2015). Effect of ligand structure on olefin polymerization by a metallocene/borate catalyst: A computational study. Organometallics.

[28] Silanes I, Mercero JM, Ugalde JM (2006). Comparison of Ti, Zr, and Hf as cations for metallocene-catalyzed olefin polymerization. Organometallics.

[29] Gaussian 16 Revision C.02; Gaussian, Inc.: Wallingford, CT (2019).

[30] Sarazin Y, Carpentier J-F (2015). Discrete cationic complexes for ring-opening polymerization catalysis of cyclic esters and epoxides. Chem. Rev..

[31] Kourti M-E, Vougioukalakis GC, Hadjichristidis N, Pitsikalis M (2011). Metallocene-mediated cationic ring-opening polymerization of 2-methyl- and 2-phenyl-oxazoline. J. Polym. Sci. A Polym. Chem..

[32] Batagianni E, Marathianos A, Koraki A, Maroudas A-P, Pitsikalis M (2016). Metallocene-mediated cationic polymerization of vinyl ethers: Kinetics of polymerization and synthesis and characterization of statistical copolymers. J Macromol. Sci. Part A Pure Appl. Chem..

[33] Villaseñor E (2013). Neutral dimethylzirconocene complexes as initiators for the ring-opening polymerization of ɛ-caprolactone. Eur. J. Inorg. Chem..

[34] Nifantev I, Ivchenko P (2019). Coordination ring-opening polymerization of cyclic esters: A critical overview of DFT modeling and visualization of the reaction mechanisms. Molecules.

[35] Nifant'ev I, Ivchenko P (2019). DFT modeling of organocatalytic ring-opening polymerization of cyclic esters: A crucial role of proton exchange and hydrogen bonding. Polymers (Basel).

[36] Meelua W, Keawkla N, Oláh J, Jitonnom J (2020). DFT study of formation and properties of dinuclear zirconocene cations: Effects of ligand structure, solvent, and metal on the dimerization process. J. Organomet. Chem..

[37] Penczek S, Cypryk M, Duda A, Kubisa P, Słomkowski S (2007). Living ring-opening polymerizations of heterocyclic monomers. Prog. Polym. Sci..

[38] Penczek S, Pretula J (2021). Activated monomer mechanism (AMM) in cationic ring-opening polymerization. The origin of the AMM and further development in polymerization of cyclic esters. ACS Macro Lett..

[39] Kohn W, Sham LJ (1965). Self-consistent equations including exchange and correlation effects. Phys. Rev..

[40] Parr RG, Yang W (1994). Density-Functional Theory of Atoms and Molecules.

[41] Nifant'ev I, Ivchenko P (2019). Coordination ring-opening polymerization of cyclic esters: A critical overview of DFT modeling and visualization of the reaction mechanisms. Molecules.

[42] Yang Y, Weaver MN, Merz KM (2009). Assessment of the "6-31+G** + LANL2DZ" mixed basis set coupled with density functional theory methods and the effective core potential: Prediction of heats of formation and ionization potentials for first-row-transition-metal complexes. J. Phys. Chem. A.

[43] Meelua W, Wanjai T, Chokbunpiam T, Jitonnom J (2023). Mechanism of ring-opening polymerization of l-lactide by lanthanide aryloxide: A theoretical study on the effect of the aryloxide ligands on the process. Int. J. Quantum Chem..

[44] Gaussian 09, Revision A.02; Gaussian, Inc.: Wallingford, CT (2009).

[45] Wang X, Lin F, Qu J, Hou Z, Luo Y (2016). DFT studies on styrene polymerization catalyzed by cationic rare-earth-metal complexes: Origin of ligand-dependent activities. Organometallics.

[46] Dapprich S, Frenking G (1995). Investigation of donor-acceptor interactions: A charge decomposition analysis using fragment molecular orbitals. J. Phys. Chem..

[47] Karttunen VA (2008). The influence of the ligand structure on activation of hafnocene polymerization catalysts: A theoretical study. J. Organomet. Chem..

[48] Karttunen VA (2008). Influence of the ligand structure of hafnocene polymerization catalysts: A theoretical study on ethene insertion and chain propagation. Organometallics.

