# Supplementary material for: Computational evaluation of zirconocene catalysts for ε-caprolactone cationic ring-opening polymerization
Source: Sci Rep. 2024 Feb 17;14:3952. doi: 10.1038/s41598-024-54157-y (PMC10874422; doi:10.1038/s41598-024-54157-y)
Supplement: Supplementary file 1 — Supplementary Information 1. [file 41598_2024_54157_MOESM1_ESM.docx]

**Supporting Information**

**Computational Evaluation of Zirconocene Catalysts for ε-Caprolactone Cationic Ring-Opening Polymerization**

Wijitra Meelua^1,2^ , Tanchanok Wanjai^2^, and Jitrayut Jitonnom ^2,^*

*^1^Demonstration School, University of Phayao, Phayao 56000, Thailand*

*^2^Unit of Excellence in Computational Molecular Science and Catalysis, and Division of Chemistry, School of Science, University of Phayao, Phayao 56000, Thailand*

| **Table of Content** | | |
| --- | --- | --- |
|  | | **Pages** |
| **I.** | Choice of DFT functional and zirconocene/cocatalyst models | S2 |
| **II.** | Comparison of computed activation energies with available experimental data | S6 |
| **III.** | Cross-correlation heatmaps | S7 |
| **IV.** | Linear correlations between the selected properties and the activation energies (Ea1) for **C^+^**, **1** and **2** | S8 |
| **V.** | Summary of data tables analyzed for 54 zirconocenes | S9 |
| **VI.** | Visualization and comparison of selected catalysts | S15 |
| **VII.** | References | S27 |

**I. Choice of DFT functional and zirconocene/cocatalyst models**

**Activation free energies.** Since the activation free energies of the CROP process will be used to compare with several parameters related to polymerization activity (see **Table S1** in Section II), we firstly test the choice of DFT functional for the study of this process by performing “gas-phase” geometry optimizations on the naked model (C^+^) of **R1** and **TS1** using different DFT/LANL2DZ+6-31G(d) methods (DFT = B3LYP-D3(BJ), M06-2X, and ωB97XD). Frequency calculations were performed at the CPCM(toluene)-DFT/6-311++G(d,p) (LANL2DZ) level on the optimized structures to obtain the activation free energies (Δ*G*^ǂ^_Ea1_) in solution for the 1^st^ propagation, which are indicated in **Figure S1** as bar plots. It is clear that B3LYP-D3(BJ) produce lower Δ*G*^ǂ^_Ea1_, compared to other DFT functional. This B3LYP-D3(BJ)/LANL2DZ method also provided the activation barriers that could account for the activity difference observed experimentally for the catalysts **C1**, **C3** and **H1** (**Table S1**). Additionally, this B3LYP-D3(BJ) functional has previously applied successfully in studying similar processes.[**^1-5^**](#_ENREF_1) Thus, the B3LYP-D3(BJ) is chosen for the current investigations.


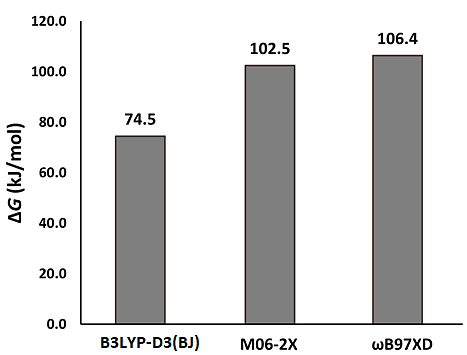


**Figure S1.** Bar chart showing the activation free energies (Δ*G*^ǂ^_Ea1_) of the 1^st^ propagation for the naked model (**C^+^**), as obtained in toluene using different DFT functional (DFT = B3LYP-D3(BJ), ωB97XD, M06-2X).

**Zirconocene/cocatalyst models.** To model the [Cp_2_ZrMe^+^][A^–^] system (A^–^ =[MeB(C_6_F_5_)_3_]^–^ (**1**) and [B(C_6_F_5_)_4_]^–^) (**2**), we first built the [(Me_5_Cp)_2_ZrMe-CL]^+^ complex structure (**H1** in **Figure 3**) with a *side* mode of CL coordination, followed by adding another monomer near the cleavage site of the first CL to form a reactant complex **R1**. This procedure is similar to our previous study on the CROP of trimethylene carbonate.[^5^](#_ENREF_5) At this **R1** stage (**Figure 2**), we added the counteranion and placed it freely in various positions around the catalytic center. After geometry optimization (at the B3LYP/LANL2DZ+6-31G(d) level), three distinct zirconocene/cocatalyst models were obtained (see **Figures S2-S3**). We then performed a relaxed scan along the C-O scissile bond of the monomer to obtain the **TS1** structures of these models, which were confirmed by vibrational frequency calculations with one imaginary value for each TS. The bar charts showing the Gibbs energy barriers calculated for the **R1**→**TS1** step of those three models are shown in **Figures S2-S3**. The results show that "position 1" with the anion close to the ring cleavage site is the preferred structure, with lower reaction barriers (~70 kJ/mol in both **1** and **2** systems).

**
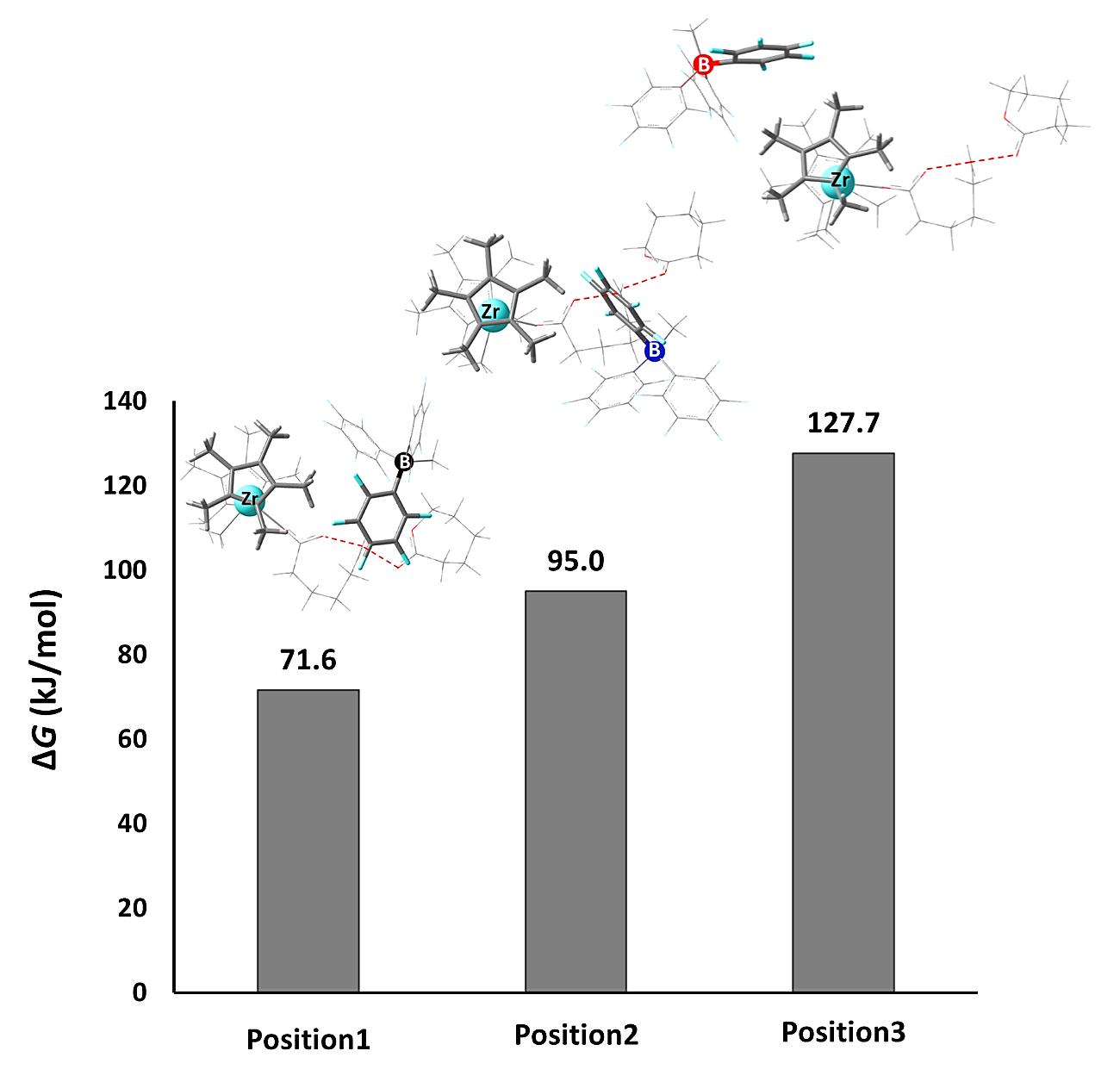
**

**Figure S2.** Bar chart showing relative Gibbs energy barriers for the 1^st^ propagation (**R1** → **TS1**) where the [MeB(C_6_F_5_)_3_]^–^ (**1**) counteranion was placed in three different positions around the catalytic center. The transition state structures were included for comparison.

**
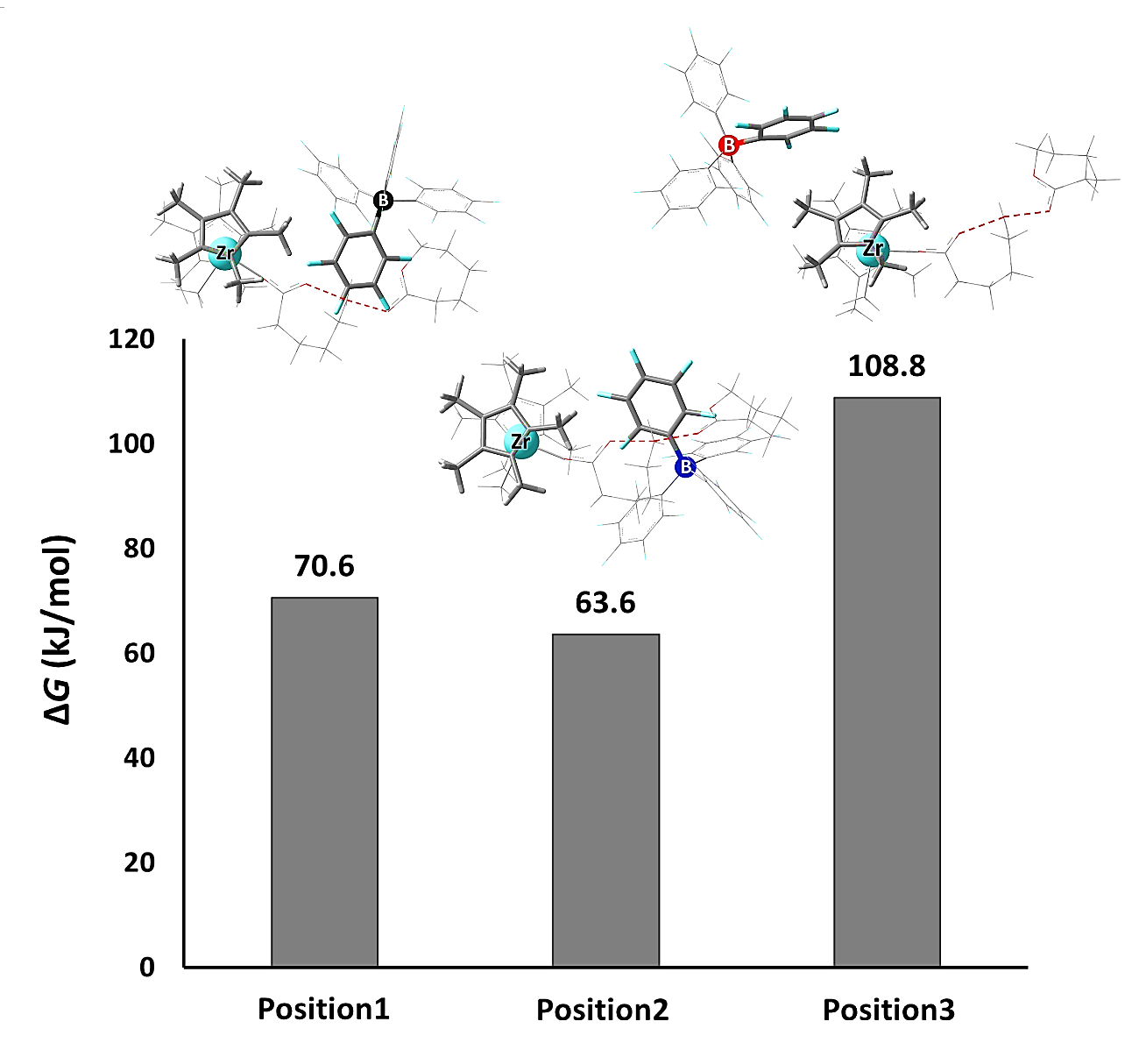
**

**Figure S3.** Bar chart showing relative Gibbs energy barriers for the 1^st^ propagation (**R1** → **TS1**) where the [B(C_6_F_5_)_4_]^–^ (**2**) counteranion was placed in three different positions around the catalytic center. The transition state structures were included for comparison.

**II. Comparison of computed activation energies with available experimental data**

**Table S1.** Comparison of computed activation energies in the 1^st^ propagation (Δ*G*^ǂ^_Ea1_) and experimental parameters (%EC, %yield, *M*_w_/*M*_n_, and *M*_n,_) reported for the polymerization activity with [Cp_2_ZrMe^+^][B(C_6_F_5_)_4_^−^] by Hayakawa et al.[^6^](#_ENREF_6) and Pitsikalis et al.[^7^](#_ENREF_7) The corresponding values using [Cp_2_ZrMe^+^][MeB(C_6_F_5_)_3_^−^] are included in parenthesis.

| **Catalyst** | **%EC** | **%Yield** | ***M*_w_/*M*_n_** | ***M*_n_**  **(g/mol)** | **Δ*G*^ǂ^_Ea1_**  **(kJ/mol)** |
| --- | --- | --- | --- | --- | --- |
| C1 | 30  (n.a.) | >90  (>95) | 1.04  (1.03) | 36,000  (30,000) | 70.6  (71.6) |
| C3 | 100  (n.a.) | n.a.  (n.a.) | 1.29  (n.a.) | 10,000  (n.a.) | 78.6  (85.7) |
| H1 | 100  (n.a.) | 88  (n.a.) | 1.31  (n.a.) | 9,200  (n.a.) | 82.3  (88.9) |
| C4 | >100  (n.a.) | > 90  (>90) | 1.16  (1.15) | 12,000  (11,000) | 83.8  (75.6) |
| H16 | >100  (n.a.) | >90  (>90) | 1.14  (1.06) | 10,000  (38,000) | 83.2  (84.5) |

n.a. not available,

%EC = %efficiency of catalyst, which is obtained using equation: %EC = (*M*_n_,_theor_ / *M*_n_,_obs_) x 100

**III. Cross-correlation heatmaps**

**
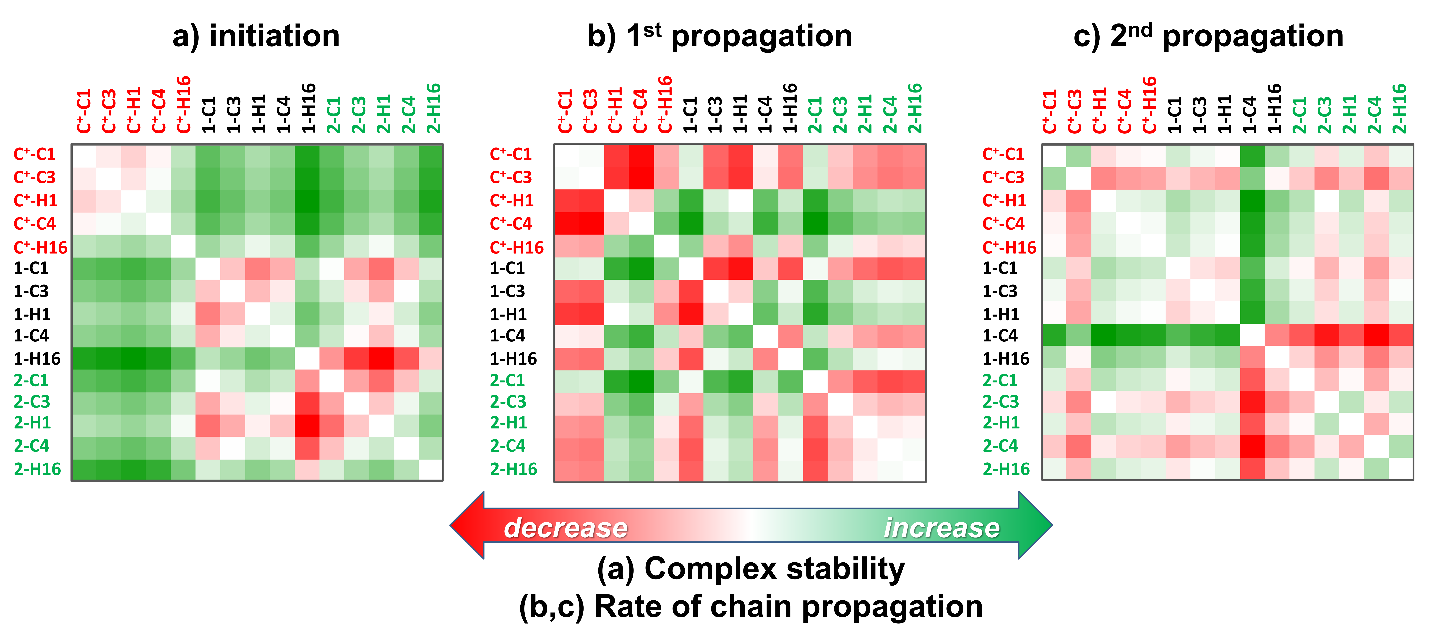
**

**Figure S4.** Cross-correlation heatmaps corresponding to the comparison of the energy difference between the pairwise of five catalyst structures (**C1**, **C3**, **H16**, **C4** and **H1**, **Figure 3**) in three catalytic systems (**C^+^**, **1** and **2**). (a) The heatmap of correlation analysis of the complexation energies for the initiation step (**Free species** → **Complex**). (b,c) The heatmap of correlation analysis of the activation energies for the 1^st^ and 2^nd^ propagations (Ea1 and Ea2, respectively). The color blocks at different positions represent the energetic comparison between the catalyst pairwise. Green and red indicate positive and negative changes that lead to increasing and decreasing of the complex stability and the rate of chain propagation, respectively. White indicates no changes due to the self-comparison.

Above is the heatmap corresponding to the energy correlations between the complexation energies and activation energies for different catalytic systems. Results in **Figure S4** clearly show that the heatmaps become gradually more complex from the initiation to the second propagation. The red and green colors on the heat maps clearly indicate that the catalysts decrease and increase the complex stability or the rate of chain propagation (via the activation barriers, Ea1 and Ea2), respectively.

**IV. Linear correlations between the selected properties and the activation energies (Ea1) for** **C^+^**, **1** **and** **2**


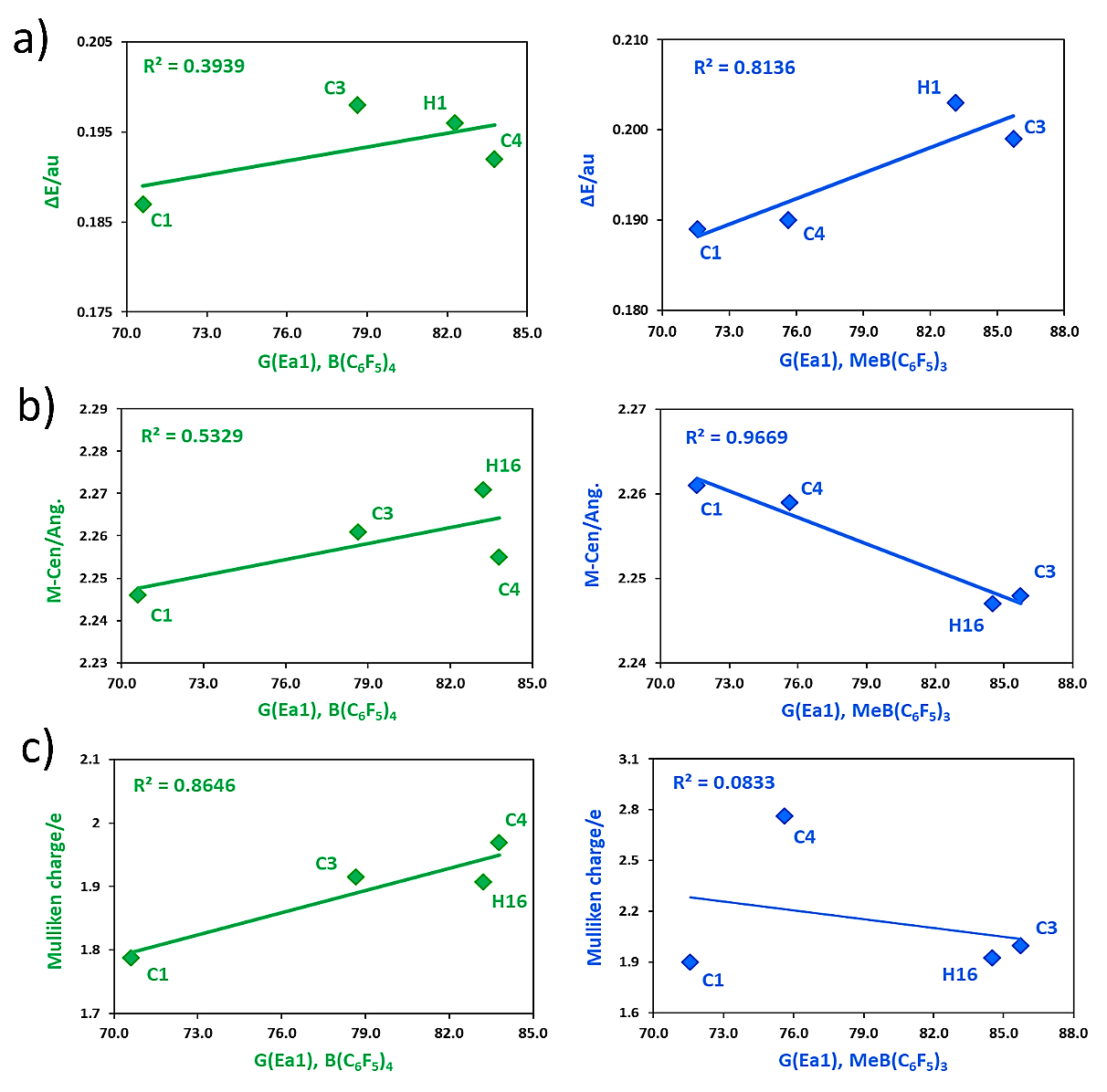


**Figure S5.** (a) Linear correlation between the Δ*E* values (au) and the activation energies (Ea1, kJ/mol). (b) Linear correlation between the M-Cen distances (Ångstrom) and the Ea1 values. c,d) Linear correlation between the Mulliken charge (in unit of *e*) on Zr atom and the Ea1 values for the two catalytic systems: MeB(C_6_F_5_)_3_ (**1**) and B(C_6_F_5_)_4_ (**2**), which were shown in blue and green, respectively. All five zirconocene catalysts (**C1**, **C3**, **H1**, **C4**, and **H16**) were considered for each system. Values of correlation coefficients (R^2^) for the relationship were also indicated in the plots.

**V. Summary of data tables analyzed for 54 zirconocenes**

**Table S2.** Selected properties (distance, angle, HOMO-LUMO, dipole, Mulliken charge) of **Cation** and **Complex** and their complexation energies (Δ*G*_com_) computed at the CPCM(toluene)-B3LYP-D3(BJ)/6-311++G(d,p) (LANL2DZ) level for different catalyst structures.





| **Catalyst** | **∆*G*_com_^a^ (kJ/mol)** | **Cation** | | | | | | | | | | **Complex** | | | | | | | | | |
| --- | --- | --- | --- | --- | --- | --- | --- | --- | --- | --- | --- | --- | --- | --- | --- | --- | --- | --- | --- | --- | --- |
|  |  | α **(^๐^)** | β **(^๐^)** | γ **(^๐^)** | δ **(^๐^)** | **D (Å)** | **M-Cen (Å)** | **RSA*^b^* (^๐^)** | **∆*E* (LUMO–HOMO) (au)** | **Dipole moment (Debye)** | **Mulliken charge, *e^-^***  **Zr / E *^c^*** | α **(^๐^)** | β **(^๐^)** | γ **(^๐^)** | δ **(^๐^)** | **D (Å)** | **M-Cen (Å)** | **RSA*^b^* (^๐^)** | **∆*E* (LUMO–HOMO) (au)** | **Dipole moment (Debye)** | **Mulliken charge (*e^-^*)**  **Zr / E *^c^*** |
| **C1** | -98.0 | 137.5 | 40.0 | - | 89.1 | 0.806 | 2.222 | -1.2 | 0.158 | 12.268 | 1.537 | 132.7 | 39.9 | - | 88.9 | 0.987 | 2.237 | -3.7 | 0.182 | 19.758 | 1.861 |
| **C2** | -70.9 | 137.1 | 43.3 | - | 89.8 | 0.805 | 2.212 | 0.2 | 0.147 | 11.764 | 1.679 | 134.28 | 49.4 | - | 89.1 | 0.873 | 2.251 | 1.8 | 0.175 | 17.151 | 2.033 |
| **C3** | -93.5 | 138.2 | 41.9 | - | 88.5 | 0.797 | 2.250 | 0.1 | 0.142 | 15.588 | 1.942 | 134.8 | 48.5 | - | 88.6 | 0.869 | 2.275 | 1.7 | 0.164 | 19.442 | 2.262 |
| **C4** | -95.7 | 135.5 | 45.8 | - | 88.5 | 0.846 | 2.208 | 0.6 | 0.160 | 16.389 | 0.996 | 132.4 | 53.8 | - | 89.7 | 0.918 | 2.248 | 3.1 | 0.173 | 11.890 | 1.062 |
| **C5** | -86.6 | 117.6 | 71.8 | 99.2 | 85.5 | 1.145 | 2.211 | 4.7 | 0.154 | 12.866 | 1.631/0.147 | 116.1 | 73.5 | 101.0 | 85.8 | 1.190 | 2.248 | 4.8 | 0.167 | 19.114 | 1.790/0.628 |
| **C6** | -94.7 | 126.6 | 58.6 | 93.0 | 87.5 | 0.998 | 2.230 | 2.6 | 0.153 | 12.017 | 1.640/0.918 | 124.2 | 61.5 | 93.9 | 87.7 | 1.057 | 2.256 | 2.8 | 0.172 | 18.799 | 1.839/0.937 |
| **C7** | -95.7 | 127.4 | 59.3 | 92.9 | 85.3 | 0.974 | 2.198 | 3.3 | 0.145 | 16.430 | 1.898/1.311 | 127.3 | 65.5 | 93.4 | 84.4 | 1.006 | 2.262 | 6.4 | 0.163 | 19.918 | 1.505/1.362 |
| **C8** | -91.1 | 128.9 | 55.8 | 90.9 | 87.3 | 0.956 | 2.205 | 2.3 | 0.144 | 16.891 | 1.912/1.247 | 127.2 | 60.2 | 91.4 | 85.6 | 1.010 | 2.265 | 3.7 | 0.163 | 19.874 | 1.447/1.286 |
| **C9** | -88.0 | 128.7 | 57.9 | 93.5 | 85.8 | 0.960 | 2.204 | 3.3 | 0.124 | 12.287 | 1.686/0.725 | 126.4 | 62.3 | 94.0 | 84.6 | 1.023 | 2.261 | 4.3 | 0.146 | 17.116 | 0.686/0.757 |
| **C10** | -87.2 | 130.0 | 54.7 | 92.1 | 87.7 | 0.934 | 2.210 | 2.4 | 0.139 | 12.497 | 1.829/1.268 | 129.5 | 59.9 | 92.4 | 85.4 | 0.971 | 2.275 | 4.7 | 0.158 | 22.230 | 1.332/1.353 |
| **C11** | -90.1 | 129.4 | 57.0 | 94.6 | 87.4 | 0.943 | 2.202 | 3.2 | 0.138 | 11.195 | 1.716/1.478 | 127.2 | 61.6 | 95.1 | 84.7 | 1.009 | 2.270 | 4.4 | 0.158 | 15.672 | 1.032/1.478 |
| **C12** | -88.8 | 130.7 | 56.3 | 94.3 | 87.2 | 0.921 | 2.199 | 3.5 | 0.139 | 13.182 | 1.642/2.004 | 129.7 | 60.5 | 94.9 | 84.9 | 0.964 | 2.271 | 5.1 | 0.158 | 17.620 | 1.055/1.806 |

**Table S2.** Cont.

| **Catalyst** | **∆*G*_com_ *^a^*  (kJ/mol)** | **Cation** | | | | | | | | | | **Complex** | | | | | | | | | |
| --- | --- | --- | --- | --- | --- | --- | --- | --- | --- | --- | --- | --- | --- | --- | --- | --- | --- | --- | --- | --- | --- |
|  |  | α **(^๐^)** | β **(^๐^)** | γ **(^๐^)** | δ **(^๐^)** | **D (Å)** | **M-Cen (Å)** | **RSA*^b^* (^๐^)** | **∆*E* (LUMO–HOMO) (au)** | **Dipole moment (Debye)** | **Mulliken charge, *e^-^***  **Zr / E *^c^*** | α **(^๐^)** | β **(^๐^)** | γ **(^๐^)** | δ **(^๐^)** | **D (Å)** | **M-Cen (Å)** | **RSA*^b^* (^๐^)** | **∆*E* (LUMO–HOMO) (au)** | **Dipole moment (Debye)** | **Mulliken charge (*e^-^*)**  **Zr / E *^c^*** |
| **C13** | -75.5 | 128.5 | 57.3 | 94.6 | 86.2 | 0.967 | 2.234 | 2.9 | 0.140 | 13.155 | 1.670/1.732 | 128.8 | 61.0 | 95.5 | 85.6 | 0.980 | 2.266 | 4.9 | 0.159 | 16.919 | 1.501/1.963 |
| **C14** | -99.8 | 138.3 | 42.1 | - | 90.0 | 0.799 | 2.247 | 0.2 | 0.151 | 1.457 | 1.610 | 133.8 | 40.7 | - | 89.1 | 0.887 | 2.260 | -2.8 | 0.174 | 14.870 | 1.719 |
| **C15** | -97.9 | 137.9 | 37.1 | - | 89.1 | 0.798 | 2.216 | -2.5 | 0.125 | 11.134 | 1.807 | 133.7 | 41.2 | - | 93.5 | 0.884 | 2.255 | -2.6 | 0.147 | 16.370 | 1.893 |
| **C16** | -90.1 | 128.1 | 56.6 | 94.0 | 86.6 | 0.965 | 2.201 | 2.3 | 0.129 | 12.700 | 1.725/1.055 | 127.3 | 61.4 | 94.4 | 84.3 | 1.005 | 2.265 | 4.3 | 0.141 | 20.848 | 1.710/1.259 |
| **C17** | -101.8 | 119.1 | 69.5 | 100.1 | 56.4 | 1.114 | 2.197 | 4.3 | 0.122 | 5.242 | 1.787/0.780 | 118.9 | 71.8 | 100.3 | 55.1 | 1.134 | 2.209 | 5.4 | 0.136 | 10.896 | 1.663/0.591 |
| **C18** | -73.9 | 129.3 | 52.3 | - | 90.5 | 0.922 | 2.138 | 1.3 | 0.127 | 16.461 | 1.780 | 127.5 | 56.4 | - | 88.1 | 0.988 | 2.234 | 1.3 | 0.136 | 22.242 | 1.509 |
| **C19** | -87.2 | 127.5 | 57.5 | - | 87.0 | 0.979 | 2.216 | 2.5 | 0.148 | 17.226 | 1.811 | 125.4 | 59.9 | - | 86.9 | 1.034 | 2.253 | 2.7 | 0.159 | 23.795 | 1.323 |
| **C20** | -92.8 | 138.0 | 42.7 | - | 93.4 | 0.818 | 2.190 | 0.3 | 0.124 | 13.974 | 1.910 | 135.8 | 51.7 | - | 87.1 | 0.863 | 2.242 | 3.7 | 0.138 | 14.860 | 2.216 |
| **C21** | -97.8 | 139.5 | 34.2 | - | 92.9 | 0.767 | 2.219 | -3.1 | 0.119 | 14.735 | 1.827 | 135.8 | 42.4 | - | 92.4 | 0.849 | 2.268 | -0.9 | 0.139 | 17.644 | 2.230 |
| **C22** | -81.0 | 119.3 | 70.0 | 102.5 | 85.7 | 1.100 | 2.185 | 4.7 | 0.113 | 13.535 | 1.793/-0.104 | 119.2 | 72.0 | 102.8 | 83.2 | 1.130 | 2.254 | 5.6 | 0.127 | 22.008 | 1.362/-0.145 |
| **C23** | -89.1 | 122.2 | 74.3 | 102.5 | 81.8 | 1.077 | 2.216 | 8.3 | 0.108 | 2.289 | 1.633/0.164 | 117.6 | 74.6 | 102.8 | 80.5 | 1.171 | 2.313 | 6.1 | 0.120 | 11.694 | 1.470/0.222 |
| **C24** | -97.9 | 119.9 | 73.8 | 99.1 | 80.3 | 1.110 | 2.185 | 6.8 | 0.117 | 8.095 | 1.853/0.248 | 119.6 | 74.7 | 99.7 | 80.3 | 1.130 | 2.254 | 7.2 | 0.125 | 20.603 | 1.347/0.397 |
| **C25** | -101.3 | 119.0 | 73.8 | 98.3 | 81.3 | 1.125 | 2.289 | 6.4 | 0.117 | 7.128 | 1.802/0.674 | 118.5 | 75.6 | 98.6 | 80.7 | 1.152 | 2.323 | 7.1 | 0.124 | 18.614 | 1.145/0.307 |
| **C26** | -97.2 | 119.7 | 70.6 | 98.9 | 81.5 | 1.101 | 2.227 | 5.2 | 0.115 | 21.051 | 1.647/0.704 | 118.1 | 74.7 | 99.7 | 80.6 | 1.160 | 2.287 | 6.4 | 0.127 | 25.668 | 1.617/0.714 |
| **C27** | -84.7 | 139.8 | 48.3 | - | 81.1 | 0.781 | 2.309 | 4.1 | 0.125 | 16.018 | 2.575 | 136.5 | 54.4 | - | 79.1 | 0.849 | 2.316 | 5.5 | 0.136 | 29.893 | 2.053 |
| **C28** | -120.0 | 128.5 | 55.8 | 95.1 | 82.2 | 0.959 | 2.285 | 2.2 | 0.133 | 45.785 | 1.576/0.636 | 131.1 | 64.1 | 95.1 | 77.4 | 0.949 | 2.338 | 7.6 | 0.127 | 30.378 | 1.059/1.608 |
| **C29** | -95.4 | 120.1 | 74.3 | 99.2 | 80.4 | 1.105 | 2.217 | 7.2 | 0.116 | 8.072 | 1.871/0.381 | 119.0 | 75.0 | 99.9 | 81.6 | 1.139 | 2.266 | 7.0 | 0.125 | 36.616 | 1.507/0.698 |
| **H1** | -87.4 | 143.0 | 37.3 | - | 91.0 | 0.719 | 2.256 | 0.2 | 0.133 | 14.711 | 2.685 | 138.8 | 40.1 | - | 90.6 | 0.804 | 2.292 | -0.5 | 0.155 | 19.544 | 2.499 |
| **H2** | -78.6 | 137.2 | 45.3 | - | 89.2 | 0.809 | 2.213 | 1.3 | 0.145 | 11.700 | 1.641 | 132.7 | 50.4 | - | 90.4 | 0.902 | 2.252 | 1.5 | 0.174 | 15.837 | 2.078 |
| **H3** | -72.2 | 128.8 | 58.3 | 93.5 | 86.3 | 0.960 | 2.221 | 3.6 | 0.146 | 12.635 | 1.799/1.111 | 128.2 | 61.6 | 94.5 | 84.3 | 0.991 | 2.271 | 4.9 | 0.164 | 16.855 | 1.716/1.308 |
| **H4** | -91.9 | 127.7 | 56.1 | 90.6 | 88.1 | 0.978 | 2.222 | 1.9 | 0.152 | 14.922 | 1.671/1.139 | 128.6 | 59.8 | 91.4 | 85.9 | 0.984 | 2.268 | 4.2 | 0.174 | 18.985 | 1.816/1.273 |
| **H5** | -98.1 | 122.8 | 64.3 | 89.7 | 87.2 | 1.068 | 2.230 | 3.6 | 0.123 | 12.812 | 1.545//0.639 | 121.8 | 64.5 | 90.7 | 86.0 | 1.094 | 2.259 | 3.2 | 0.149 | 17.361 | 1.625/0.785 |

**Table S2.** Cont.

| **Catalyst** | **∆*G*_com_ *^a^*  (kJ/mol)** | **Cation** | | | | | | | | | | **Complex** | | | | | | | | | |
| --- | --- | --- | --- | --- | --- | --- | --- | --- | --- | --- | --- | --- | --- | --- | --- | --- | --- | --- | --- | --- | --- |
|  |  | α **(^๐^)** | β **(^๐^)** | γ **(^๐^)** | δ **(^๐^)** | **D (Å)** | **M-Cen (Å)** | **RSA*^b^* (^๐^)** | **∆*E* (LUMO–HOMO) (au)** | **Dipole moment (Debye)** | **Mulliken charge, *e^-^***  **Zr / E *^c^*** | α **(^๐^)** | β **(^๐^)** | γ **(^๐^)** | δ **(^๐^)** | **D (Å)** | **M-Cen (Å)** | **RSA*^b^* (^๐^)** | **∆*E* (LUMO–HOMO) (au)** | **Dipole moment (Debye)** | **Mulliken charge (*e^-^*)**  **Zr / E *^c^*** |
| **H6** | -97.8 | 124.2 | 64.5 | 89.6 | 86.6 | 1.046 | 2.240 | 4.4 | 0.146 | 13.512 | 1.541/0.462 | 123.2 | 65.2 | 90.5 | 85.7 | 1.076 | 2.262 | 4.2 | 0.168 | 20.301 | 1.549/0.841 |
| **H7** | -83.0 | 140.0 | 36.9 | - | 93.2 | 0.755 | 2.191 | -1.6 | 0.129 | 16.282 | 2.178 | 136.8 | 45.7 | - | 88.3 | 0.837 | 2.281 | 1.2 | 0.148 | 25.146 | 2.111 |
| **H8** | -88.0 | 128.8 | 56.2 | 94.4 | 86.2 | 0.951 | 2.206 | 2.5 | 0.128 | 14.104 | 1.500/1.338 | 127.3 | 61.4 | 94.4 | 83.7 | 1.005 | 2.288 | 4.3 | 0.140 | 15.352 | 1.102/1.518 |
| **H9** | -104.1 | 138.1 | 40.0 | - | 87.4 | 0.798 | 2.242 | -1.0 | 0.128 | 9.821 | 1.647 | 133.6 | 40.4 | - | 92.0 | 0.886 | 2.252 | -3.0 | 0.148 | 14.614 | 2.153 |
| **H10** | -126.8 | 138.2 | 38.4 | - | 92.2 | 0.807 | 2.297 | -1.7 | 0.127 | 17.172 | 1.430 | 132.7 | 46.1 | - | 92.0 | 0.910 | 2.299 | -0.6 | 0.143 | 14.242 | 1.936 |
| **H11** | -108.1 | 142.9 | 31.4 | - | 77.0 | 0.730 | 2.344 | -2.9 | 0.125 | 14.984 | 1.699 | 137.7 | 39.7 | - | 71.8 | 0.829 | 2.336 | -1.3 | 0.144 | 12.590 | 2.266 |
| **H12** | -119.3 | 138.2 | 40.5 | - | 92.5 | 0.811 | 2.309 | -0.7 | 0.128 | 15.204 | 1.281 | 133.2 | 47.4 | - | 91.7 | 0.903 | 2.302 | 0.3 | 0.144 | 12.956 | 1.855 |
| **H13** | -91.0 | 127.5 | 59.1 | 94.5 | 81.9 | 0.990 | 2.259 | 3.3 | 0.127 | 17.315 | 1.756/1.232 | 127.8 | 61.4 | 94.5 | 80.1 | 0.992 | 2.330 | 4.6 | 0.126 | 16.396 | 1.614/1.339 |
| **H14** | -139.3 | 137.6 | 41.3 | - | 89.7 | 0.811 | 2.234 | -0.6 | 0.142 | 19.503 | 1.606 | 133.6 | 42.6 | - | 87.0 | 0.886 | 2.250 | -1.9 | 0.143 | 19.972 | 2.640 |
| **H15** | -126.0 | 139.2 | 40.5 | - | 88.6 | 0.777 | 2.235 | -0.2 | 0.136 | 15.772 | 1.495 | 133.5 | 53.8 | - | 82.5 | 0.907 | 2.318 | 3.7 | 0.144 | 17.629 | 2.816 |
| **H16** | -122.3 | 139.0 | 42.1 | - | 93.2 | 0.792 | 2.255 | 0.6 | 0.138 | 16.600 | 1.672 | 132.2 | 50.1 | - | 83.9 | 0.925 | 2.294 | 1.2 | 0.141 | 19.190 | 2.007 |
| **H17** | -85.1 | 136.3 | 45.8 | - | 87.2 | 0.826 | 2.219 | 1.0 | 0.151 | 17.404 | 1.802 | 135.5 | 50.8 | - | 83.9 | 0.861 | 2.276 | 3.2 | 0.166 | 22.217 | 2.058 |
| **H18** | -96.5 | 130.1 | 55.2 | 94.0 | 87.8 | 0.920 | 2.175 | 2.6 | 0.118 | 1.898 | 1.732/1.221 | 125.4 | 63.2 | 94.9 | 83.7 | 1.046 | 2.283 | 4.3 | 0.130 | 16.785 | 1.324/1.465 |
| **H19** | -95.3 | 135.4 | 37.6 | - | 95.2 | 0.840 | 2.222 | -3.5 | 0.125 | 17.400 | 1.624 | 131.5 | 49.8 | - | 83.7 | 0.932 | 2.283 | 0.6 | 0.130 | 19.427 | 2.008 |
| **H20** | -114.4 | 141.8 | 35.6 | - | 88.3 | 0.728 | 2.247 | -1.3 | 0.125 | 18.009 | 1.892 | 132.9 | 51.1 | - | 82.6 | 0.922 | 2.301 | 2.0 | 0.133 | 18.950 | 1.964 |
| **H21** | -99.7 | 137.7 | 43.7 | - | 82.8 | 0.817 | 2.305 | 0.7 | 0.127 | 19.186 | 1.736 | 133.0 | 46.6 | - | 82.0 | 0.905 | 2.299 | -0.2 | 0.137 | 16.736 | 2.257 |
| **H22** | -127.3 | 139.1 | 43.7 | - | 86.9 | 0.792 | 2.226 | 1.4 | 0.137 | 7.566 | 1.647 | 136.4 | 55.0 | - | 78.3 | 0.840 | 2.266 | 5.7 | 0.133 | 20.762 | 1.886 |
| **H23** | -106.1 | 129.1 | 59.8 | 94.7 | 83.2 | 0.969 | 2.317 | 4.5 | 0.133 | 6.781 | 1.638/1.311 | 128.1 | 64.7 | 94.6 | 78.9 | 0.999 | 2.303 | 6.4 | 0.131 | 18.440 | 1.341/1.225 |
| **H24** | -102.3 | 137.6 | 54.6 | - | 78.1 | 0.797 | 2.335 | 6.1 | 0.136 | 8.382 | 2.261 | 139.2 | 42.3 | - | 81.2 | 0.828 | 2.355 | 0.8 | 0.138 | 18.248 | 2.031 |
| **H25** | -103.1 | 129.6 | 59.4 | 95.9 | 83.0 | 0.967 | 2.344 | 4.5 | 0.130 | 7.269 | 1.857/1.581 | 130.2 | 66.1 | 95.4 | 77.9 | 0.967 | 2.355 | 8.2 | 0.132 | 17.898 | 1.308/1.423 |

*^a^* **∆*G***_com_ are obtained as a difference between the Gibbs energies of the **Complex** and **Cation** from the equation: Cp_2_ZrMe^+^ + CL → [Cp_2_ZrMe-CL]^+^ + **∆*G***. *^b^* RSA = $\left( \text{ }\frac{\text{β}\text{ }\text{+}\text{ }\text{α}\text{ }}{\text{2}} \right)\text{- 90}$, *^c^* E = C/Si/Ge/P

**Table S3.** Activation energies (Ea1) and selected properties (distance, angle, HOMO-LUMO, dipole, Mulliken charge) along the first propagation (**R1** and **TS1**) for different catalyst structures. The values of Ea1 were obtained at the CPCM(toluene)-B3LYP-D3(BJ)/6-311++G(d,p) (LANL2DZ) level of theory.

| **Catalyst** | **Ea1**  **(kJ/mol)** | **First propagation** | | | | | | | **∆*E* (LUMO–HOMO) (au)** | | **Dipole moment (Debye)** | | **Mulliken charge (*e^-^*) on Zr/E** | |
| --- | --- | --- | --- | --- | --- | --- | --- | --- | --- | --- | --- | --- | --- | --- |
|  |  | α  (°) | β  (°) | γ  (°) | δ  (°) | **D**  **(Å)** | **M-Cen**  **(Å)** | **RSA**  (°) | **R1** | **TS1** | **R1** | **TS1** | **R1** | **TS1** |
| **C1** | 74.5 | 132.8 | 39.1 | - | 89.4 | 0.903 | 2.260 | -4.0 | 0.184 | 0.184 | 21.203 | 33.273 | 1.847 | 1.858 |
| **C2** | 73.1 | 131.5 | 50.2 | - | 90.0 | 0.927 | 2.265 | 0.9 | 0.176 | 0.180 | 23.350 | 25.619 | 2.040 | 2.003 |
| **C3** | 74.0 | 133.5 | 46.7 | - | 89.4 | 0.897 | 2.275 | 0.1 | 0.165 | 0.166 | 24.867 | 28.519 | 2.135 | 2.111 |
| **C4** | 92.5 | 133.3 | 48.8 | - | 90.1 | 0.905 | 2.276 | 1.1 | 0.169 | 0.167 | 18.269 | 23.851 | 2.177 | 2.093 |
| **C5** | 76.9 | 113.3 | 72.7 | 100.6 | 86.3 | 1.234 | 2.259 | 3.0 | 0.168 | 0.171 | 21.124 | 28.089 | 1.953/0.681 | 1.717/0.639 |
| **C6** | 76.9 | 124.8 | 55.5 | 97.4 | 89.5 | 1.059 | 2.272 | 0.2 | 0.177 | 0.179 | 20.753 | 28.149 | 1.974/0.901 | 1.765/0.916 |
| **C7** | 84.0 | 125.6 | 65.0 | 94.0 | 84.4 | 1.039 | 2.276 | 5.3 | 0.165 | 0.165 | 22.744 | 27.637 | 1.507/1.362 | 1.594/1.371 |
| **C8** | 85.4 | 127.1 | 60.2 | 92.2 | 85.6 | 1.016 | 2.278 | 3.6 | 0.165 | 0.166 | 25.508 | 33.184 | 1.482/1.323 | 1.571/1.299 |
| **C9** | 82.5 | 125.4 | 62.0 | 94.8 | 85.0 | 1.044 | 2.276 | 3.7 | 0.148 | 0.153 | 23.730 | 25.883 | 0.975/0.783 | 0.850/0.817 |
| **C10** | 99.5 | 125.4 | 59.9 | 93.1 | 86.9 | 1.045 | 2.282 | 2.6 | 0.160 | 0.158 | 28.799 | 26.822 | 1.364/1.362 | 1.516/1.372 |
| **C11** | 82.0 | 120.7 | 60.6 | 95.9 | 89.5 | 1.123 | 2.264 | 0.6 | 0.160 | 0.160 | 24.557 | 33.050 | 1.277/1.341 | 1.192/1.492 |
| **C12** | 86.2 | 127.3 | 61.3 | 95.3 | 86.3 | 1.010 | 2.267 | 4.3 | 0.160 | 0.159 | 20.269 | 24.107 | 1.551/1.930 | 1.766/1.801 |
| **C13** | 83.2 | 128.2 | 61.8 | 96.3 | 84.8 | 0.998 | 2.285 | 5.0 | 0.160 | 0.159 | 20.724 | 28.764 | 1.413/1.999 | 1.566/1.808 |
| **C14** | 79.5 | 132.8 | 38.6 | - | 88.4 | 0.899 | 2.246 | -4.3 | 0.175 | 0.178 | 18.848 | 27.940 | 1.713 | 1.691 |
| **C15** | 77.4 | 133.2 | 40.1 | - | 93.2 | 0.897 | 2.264 | -3.4 | 0.147 | 0.154 | 18.999 | 23.365 | 1.918 | 2.016 |
| **C16** | 74.1 | 125.6 | 61.6 | 95.2 | 84.3 | 1.039 | 2.282 | 3.6 | 0.143 | 0.147 | 18.878 | 23.385 | 1.860/1.237 | 1.764/1.296 |
| **C17** | 83.8 | 116.7 | 73.9 | 100.8 | 86.3 | 1.188 | 2.244 | 5.3 | 0.138 | 0.142 | 16.526 | 23.092 | 1.689/0.605 | 1.447/0.561 |
| **C18** | 85.4 | 125.6 | 57.2 | - | 88.3 | 1.028 | 2.256 | 1.4 | 0.136 | 0.138 | 26.851 | 29.897 | 1.483 | 1.454 |
| **C19** | 82.6 | 124.9 | 58.8 | - | 87.2 | 0.899 | 2.261 | 1.9 | 0.164 | 0.166 | 24.838 | 29.585 | 1.361 | 1.240 |
| **C20** | 78.9 | 135.7 | 50.7 | - | 87.0 | 0.866 | 2.263 | 3.2 | 0.140 | 0.184 | 19.259 | 35.133 | 2.177 | 2.031 |

**Table S3.** Cont.

| **Catalyst** | **Ea1**  **(kJ/mol)** | **First propagation** | | | | | | | **∆*E* (LUMO–HOMO) (au)** | | **Dipole moment (Debye)** | | **Mulliken charge (*e^-^*) on Zr/E** | |
| --- | --- | --- | --- | --- | --- | --- | --- | --- | --- | --- | --- | --- | --- | --- |
|  |  | α  (°) | β  (°) | γ  (°) | δ  (°) | **D**  **(Å)** | **M-Cen**  **(Å)** | **RSA**  (°) | **R1** | **TS1** | **R1** | **TS1** | **R1** | **TS1** |
| **C21** | 90.5 | 133.9 | 39.8 | - | 87.2 | 0.898 | 2.300 | -3.1 | 0.140 | 0.145 | 21.591 | 25.237 | 2.215 | 1.482 |
| **C22** | 80.3 | 116.9 | 72.9 | 103.3 | 84.0 | 1.184 | 2.288 | 4.9 | 0.127 | 0.139 | 25.847 | 33.570 | 1.330/-0.154 | 1.248/-0.131 |
| **C23** | 79.4 | 118.6 | 83.2 | 103.6 | 78.4 | 1.204 | 2.361 | 10.9 | 0.121 | 0.131 | 24.617 | 33.693 | 1.205/0.219 | 0.995/0.232 |
| **C24** | 81.0 | 115.3 | 74.4 | 100.3 | 82.2 | 1.200 | 2.288 | 4.9 | 0.125 | 0.123 | 24.345 | 33.109 | 1.380/0.442 | 1.305/0.430 |
| **C25** | 79.4 | 114.1 | 75.7 | 99.2 | 80.2 | 1.230 | 2.363 | 4.9 | 0.125 | 0.130 | 24.428 | 32.453 | 1.094/0.335 | 1.075/0.209 |
| **C26** | 85.1 | 118.8 | 75.5 | 100.2 | 79.5 | 1.159 | 2.322 | 7.2 | 0.128 | 0.129 | 33.117 | 41.973 | 1.731/0.706 | 1.468/0.726 |
| **C27** | 90.5 | 136.5 | 55.2 | - | 77.4 | 0.864 | 2.385 | 5.9 | 0.141 | 0.130 | 33.032 | 43.237 | 2.243 | 2.095 |
| **C28** | 85.2 | 126.5 | 66.4 | 97.3 | 78.6 | 1.044 | 2.360 | 6.5 | 0.130 | 0.144 | 28.561 | 21.067 | 1.225/1.520 | 1.144/1.481 |
| **C29** | 78.8 | 118.3 | 75.6 | 100.5 | 80.3 | 1.162 | 2.304 | 6.9 | 0.126 | 0.135 | 25.493 | 20.887 | 1.535/0.678 | 1.406/0.638 |
| **H1** | 88.9 | 139.2 | 40.9 | - | 90.1 | 0.802 | 2.308 | 0.0 | 0.158 | 0.131 | 23.364 | 28.251 | 2.508 | 2.500 |
| **H2** | 75.0 | 132.3 | 50.3 | - | 89.1 | 0.913 | 2.265 | 1.3 | 0.175 | 0.158 | 22.980 | 24.604 | 1.933 | 1.905 |
| **H3** | 69.9 | 126.1 | 61.7 | 95.3 | 85.4 | 1.032 | 2.281 | 3.9 | 0.168 | 0.179 | 22.682 | 26.581 | 1.800/1.289 | 1.295/1.440 |
| **H4** | 81.7 | 125.4 | 58.5 | 92.2 | 87.0 | 1.042 | 2.274 | 1.9 | 0.177 | 0.169 | 20.615 | 27.295 | 1.884/1.280 | 1.696/1.291 |
| **H5** | 73.5 | 120.9 | 63.7 | 91.5 | 85.6 | 1.118 | 2.271 | 2.3 | 0.151 | 0.179 | 23.570 | 25.861 | 1.687/0.754 | 1.595/0.583 |
| **H6** | 78.1 | 121.6 | 65.7 | 91.2 | 84.9 | 1.126 | 2.275 | 3.6 | 0.170 | 0.162 | 24.026 | 26.339 | 1.652/0.854 | 1.588/0.821 |
| **H7** | 88.9 | 134.5 | 46.9 | - | 87.8 | 0.882 | 2.290 | 0.7 | 0.152 | 0.175 | 18.856 | 27.007 | 2.129 | 1.842 |
| **H8** | 83.0 | 126.6 | 61.0 | 95.4 | 85.3 | 1.021 | 2.288 | 3.8 | 0.140 | 0.150 | 18.415 | 26.766 | 1.076/1.608 | 0.979/1.478 |
| **H9** | 76.5 | 133.2 | 44.0 | - | 88.7 | 0.893 | 2.249 | -1.4 | 0.149 | 0.145 | 17.191 | 24.320 | 2.164 | 2.059 |
| **H10** | 82.3 | 133.4 | 47.6 | - | 90.6 | 0.903 | 2.319 | 0.5 | 0.143 | 0.152 | 17.041 | 25.714 | 1.965 | 1.812 |
| **H11** | 83.5 | 136.3 | 39.8 | - | 92.0 | 0.860 | 2.352 | -1.9 | 0.144 | 0.147 | 15.744 | 25.453 | 2.250 | 2.130 |
| **H12** | 85.9 | 134.1 | 48.1 | - | 91.0 | 0.892 | 2.321 | 1.1 | 0.145 | 0.146 | 16.035 | 24.356 | 1.917 | 1.633 |
| **H13** | 79.9 | 126.5 | 65.4 | 95.3 | 80.4 | 1.034 | 2.350 | 5.9 | 0.127 | 0.147 | 18.464 | 26.607 | 1.694/1.338 | 1.548/1.346 |
| **H14** | 85.8 | 133.8 | 43.4 | - | 86.2 | 0.886 | 2.272 | -1.4 | 0.149 | 0.146 | 22.584 | 25.087 | 2.482 | 2.419 |
| **H15** | 86.9 | 132.4 | 54.7 | - | 82.5 | 0.934 | 2.346 | 3.5 | 0.147 | 0.142 | 20.675 | 24.799 | 2.571 | 2.708 |

**Table S3.** Cont.

| **Catalyst** | **Ea1**  **(kJ/mol)** | **First propagation** | | | | | | | **∆*E* (LUMO–HOMO) (au)** | | **Dipole moment (Debye)** | | **Mulliken charge (*e^-^*) on Zr/E** | |
| --- | --- | --- | --- | --- | --- | --- | --- | --- | --- | --- | --- | --- | --- | --- |
|  |  | α  (°) | β  (°) | γ  (°) | δ  (°) | **D**  **(Å)** | **M-Cen**  **(Å)** | **RSA**  (°) | **R1** | **TS1** | **R1** | **TS1** | **R1** | **TS1** |
| **H16** | 80.7 | 131.1 | 36.8 | - | 88.6 | 0.935 | 2.244 | -6.1 | 0.142 | 0.151 | 25.798 | 29.437 | 1.989 | 1.804 |
| **H17** | 79.9 | 133.0 | 48.2 | - | 85.9 | 0.901 | 2.267 | 0.6 | 0.170 | 0.172 | 24.327 | 29.147 | 2.126 | 2.109 |
| **H18** | 81.2 | 120.5 | 61.1 | 95.8 | 89.5 | 1.127 | 2.271 | 0.8 | 0.131 | 0.137 | 19.518 | 23.580 | 1.279/1.419 | 1.159/1.627 |
| **H19** | 70.1 | 132.1 | 49.3 | - | 82.4 | 0.931 | 2.313 | 0.7 | 0.133 | 0.138 | 20.602 | 25.911 | 2.019 | 2.022 |
| **H20** | 89.4 | 132.3 | 50.4 | - | 82.7 | 0.936 | 2.308 | 1.3 | 0.135 | 0.142 | 23.543 | 25.226 | 1.913 | 1.790 |
| **H21** | 69.4 | 131.2 | 46.1 | - | 82.7 | 0.943 | 2.314 | -1.3 | 0.138 | 0.142 | 20.818 | 25.975 | 2.296 | 2.151 |
| **H22** | 80.8 | 134.8 | 55.3 | - | 78.7 | 0.875 | 2.288 | 5.0 | 0.136 | 0.141 | 24.752 | 34.518 | 1.883 | 1.851 |
| **H23** | 83.8 | 128.3 | 65.7 | 95.5 | 77.7 | 1.005 | 2.321 | 7.0 | 0.131 | 0.136 | 24.170 | 33.630 | 1.304/1.241 | 1.219/1.278 |
| **H24** | 73.4 | 136.6 | 55.7 | - | 76.0 | 0.860 | 2.408 | 6.2 | 0.140 | 0.144 | 24.656 | 34.289 | 2.090 | 1.904 |
| **H25** | 86.5 | 129.2 | 67.8 | 96.1 | 76.3 | 0.995 | 2.405 | 8.5 | 0.133 | 0.137 | 24.986 | 34.734 | 1.307/1.429 | 1.075/1.620 |

*^a^* Ea1 values are obtained as a difference between the Gibbs energies of the **R1** and **TS1**. *^b^* RSA = $\left( \text{ }\frac{\text{β}\text{ }\text{+}\text{ }\text{α}\text{ }}{\text{2}} \right)\text{- 90}$, *^c^* E = C/Si/Ge/P

VI. Visualization and comparison of selected catalysts


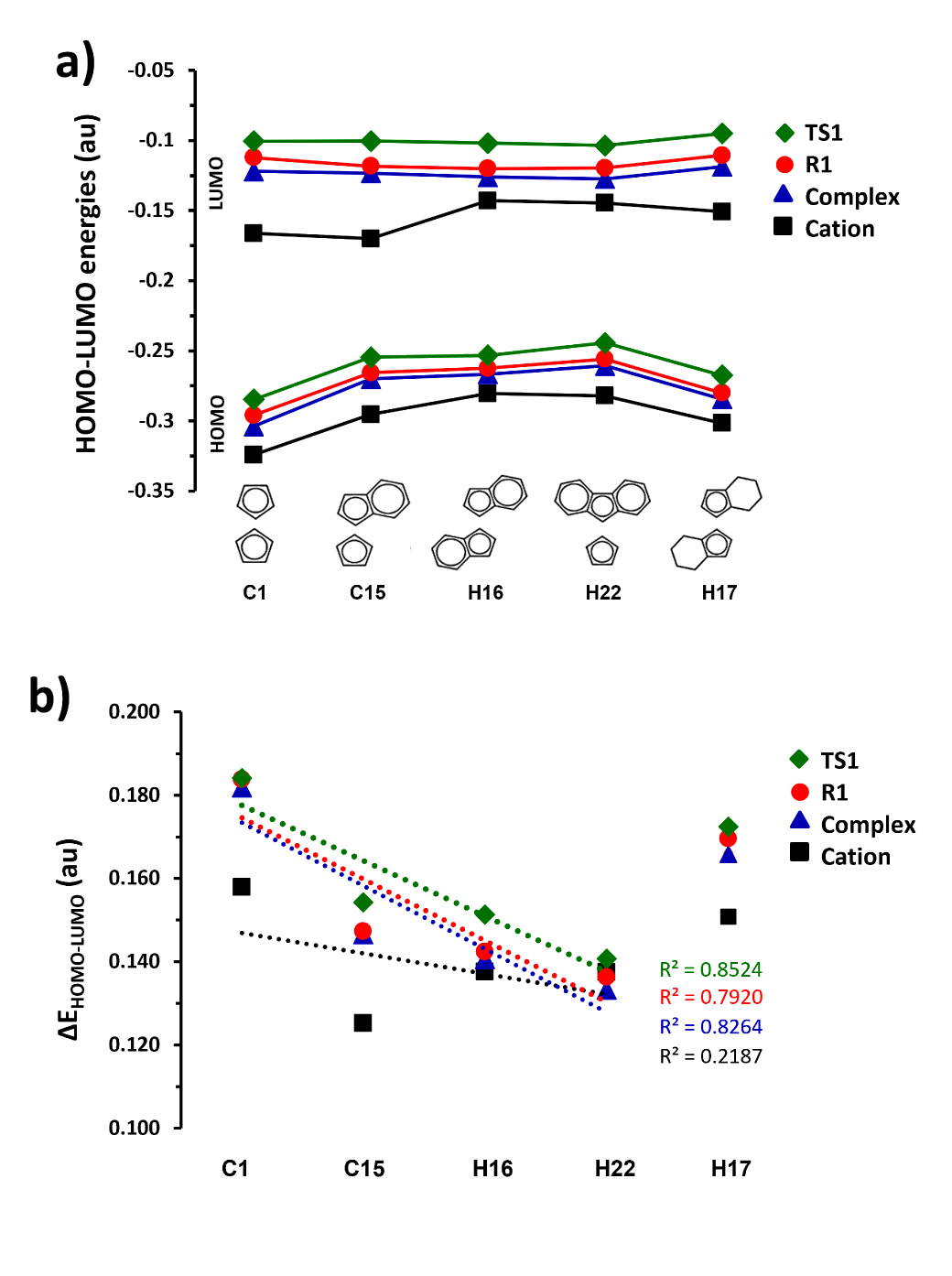


Figure S6. (a) HOMO-LUMO energies of intermediate species (Cation, Complex, R1, and TS1) for C1, C15, H16, H22, and H17. (b) Relationship between the ∆*E*_HOMO-LUMO_ values in different ancillary ligands.

**
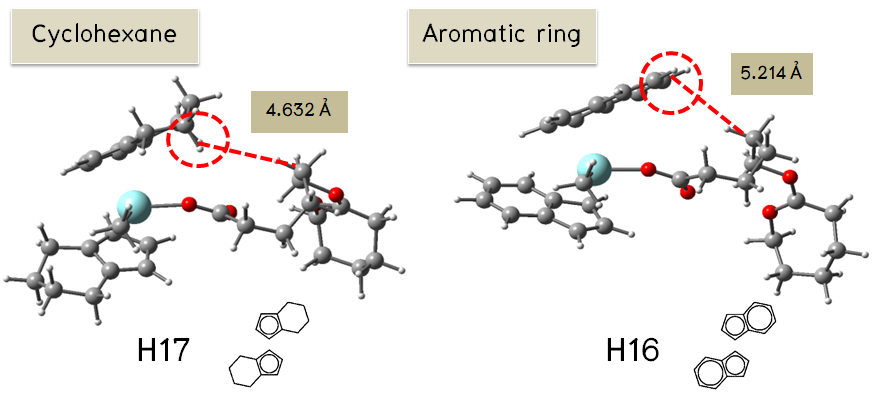
**

Figure S7. Steric interaction between the cyclohexane ring of the ligand and the
-CH_2_- group of the monomer, as found for H16 and H17.

*
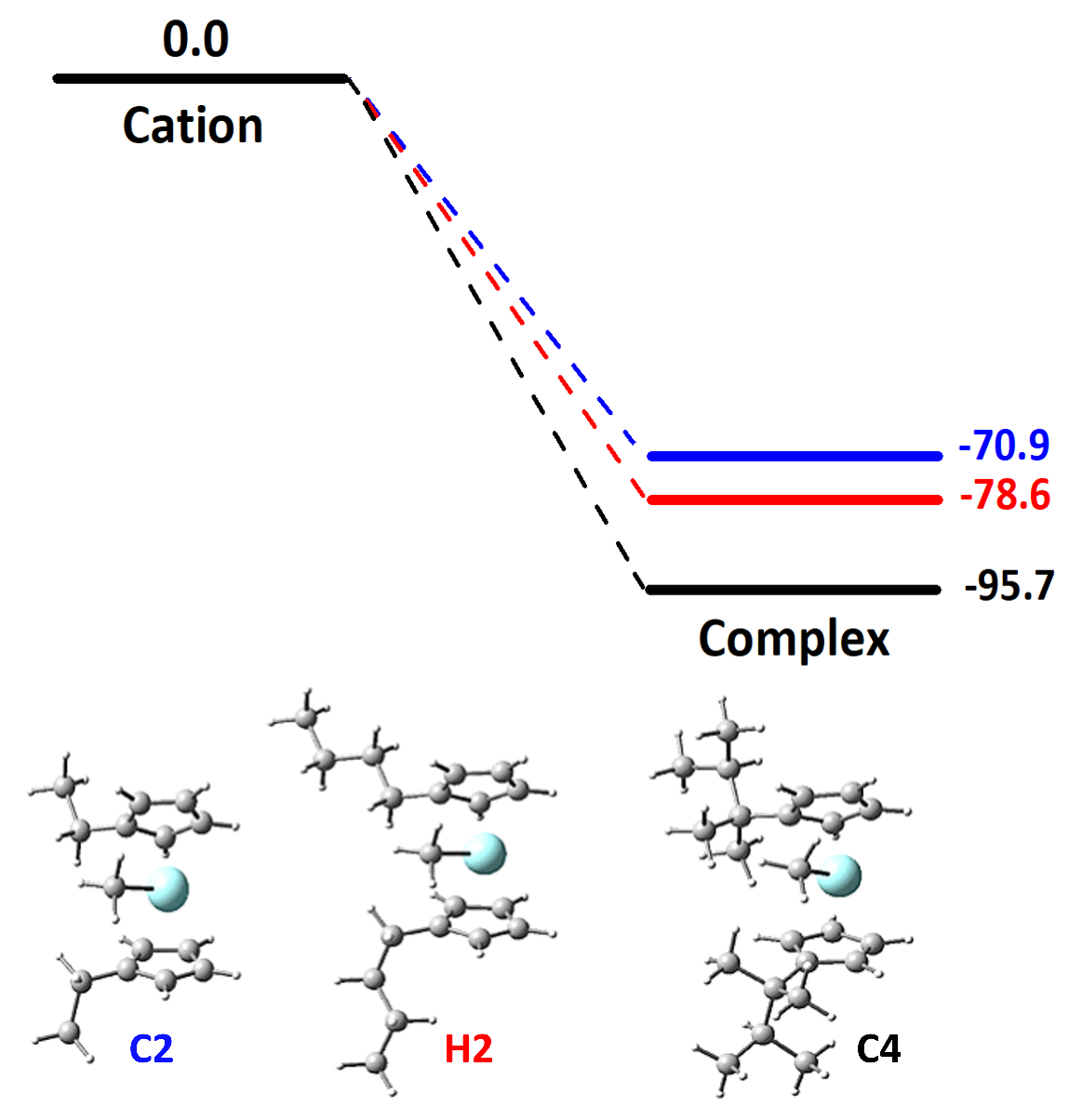
*

Figure S8. Relative energies (kJ/mol) of the initiation/complexation step for C2, H2, and C4.

*
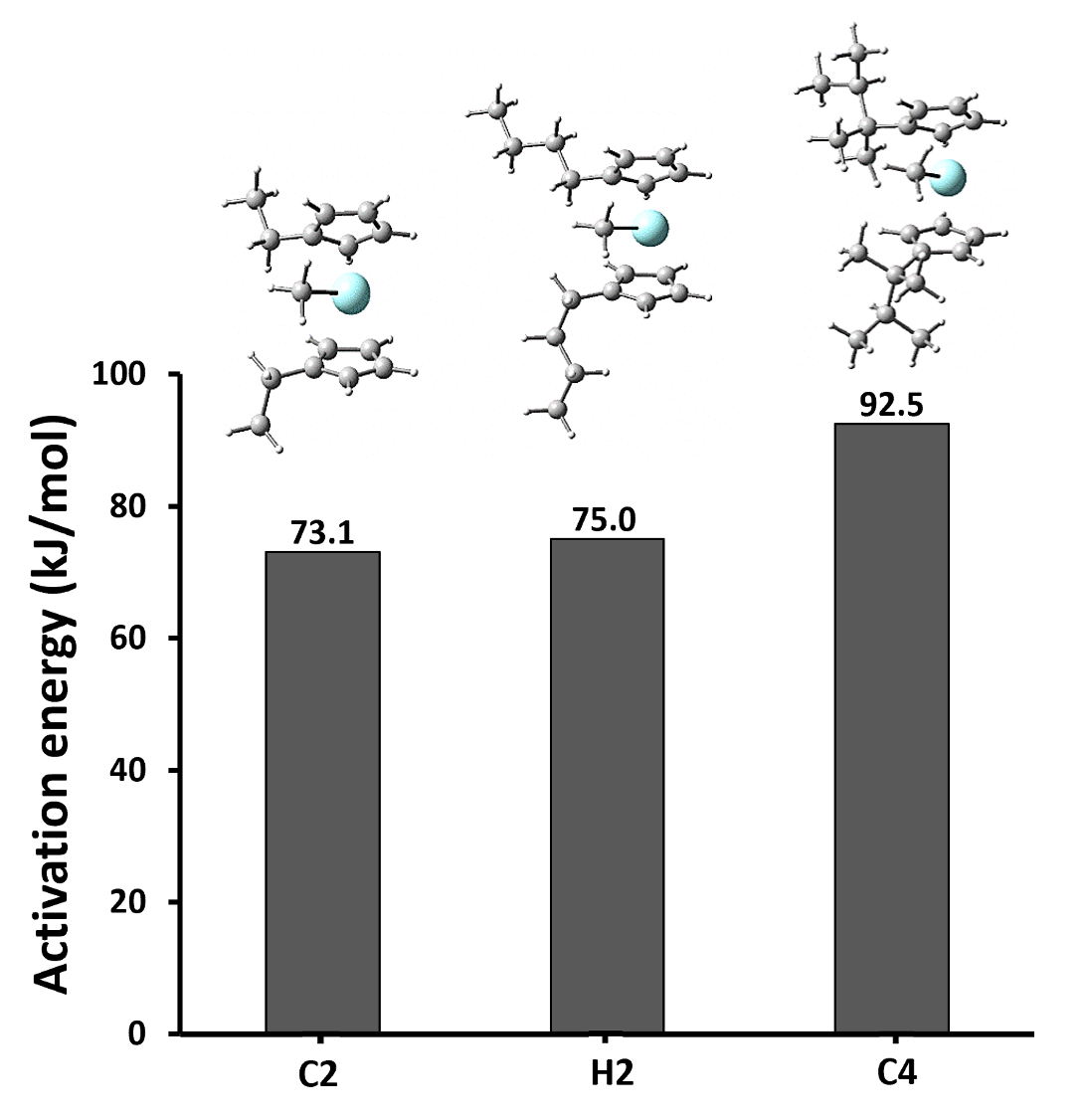
*

Figure S9. Activation energies (kJ/mol) of the first propagation step for C2, H2, and C4.

***- Conformational search: the case of C2***

Regarding the alkyl substituent discussed in the main text, this type of modification can create different conformers (with respect to the coordination of monomer) in the initiation stage for a set of ligand studied. For a simple case, C2 is chosen for this demonstration. It was found that four possible conformations, namely, C2[1,2’], C2[1,3’], C2[1,4’], C2[1,5’] were obtained during the geometry optimizations and their relative stabilities differ slightly, due to the degree of steric hindrance on the Cp’ ring with respect to the monomer. The calculations showed the relative stabilities of these conformers as C2[1,3’] < C2[1,2’] < C2[1,5’] < C2[1,4’] (Figure S10). Thus, C2[1,4’] is relatively stable than the other conformers. The least stable Complex in C2[1,3’] arises from the steric unfavourable interaction between the alkyl group and the monomer (Figure S11). Geometry optimization starting with C2[1,1’] (0 deg) geometry was also performed at the Cation and its stable conformer could not be located due to the strong repulsion between the alkyl groups between the two Cp rings, as shown in Figure S12.


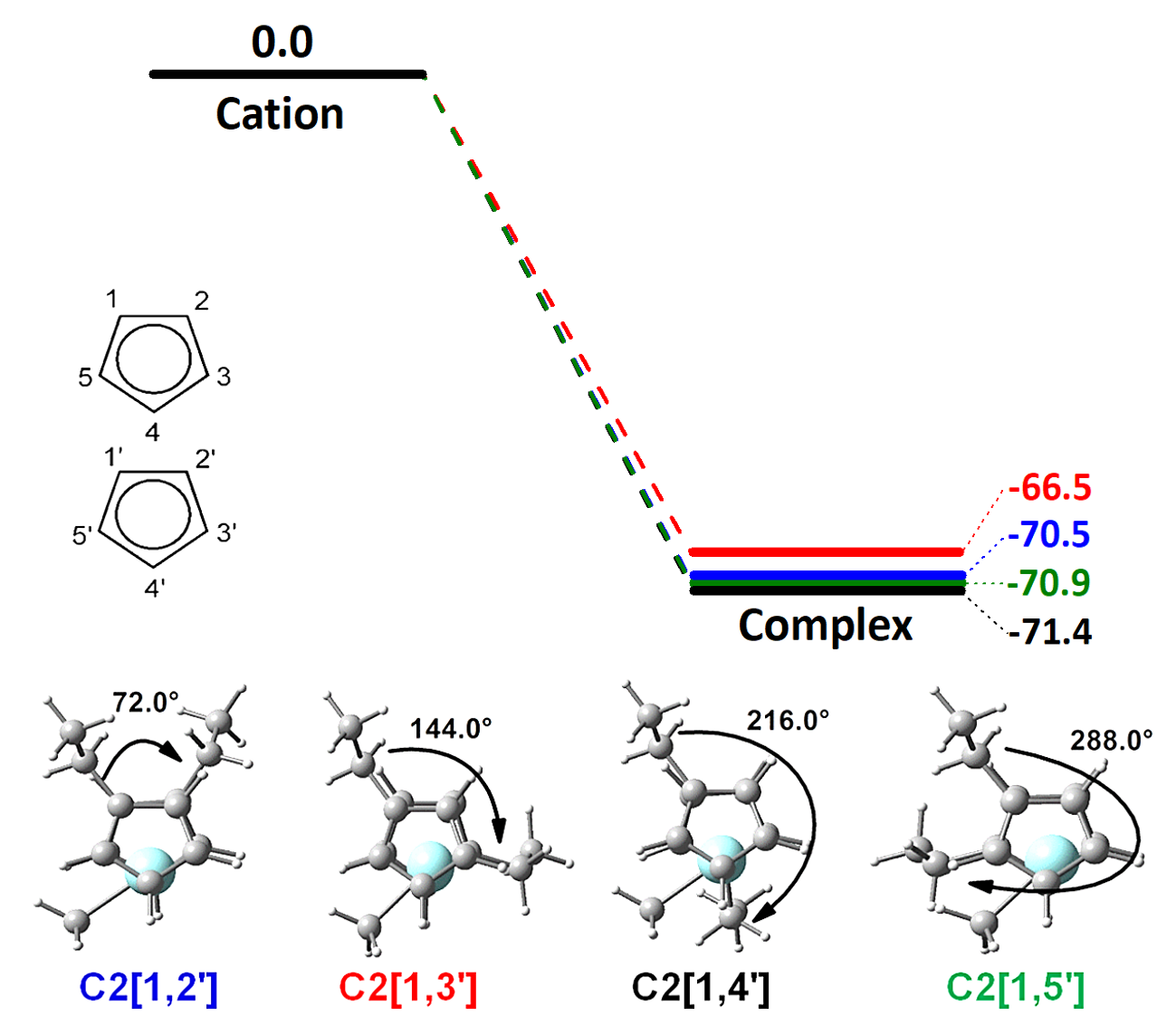
Figure S10. Relative energies (kJ/mol) of the initiation step for C2 in different conformers.


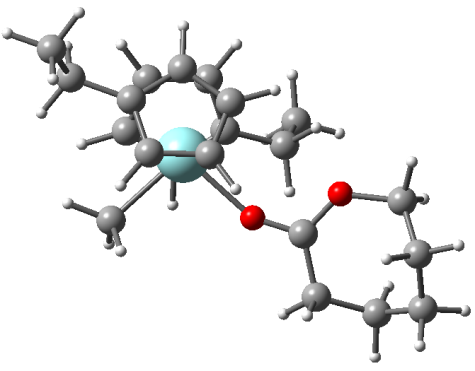

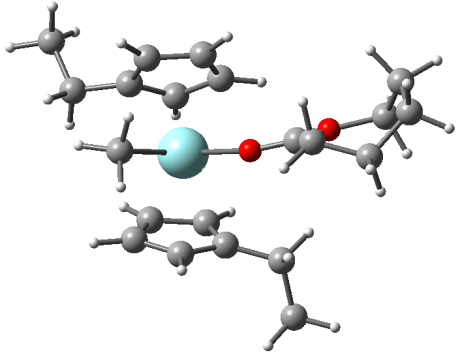


**Top view**

**Front view**

Figure S11. Optimized structures of C2 (144°)/C2[1,3’] at Complex.


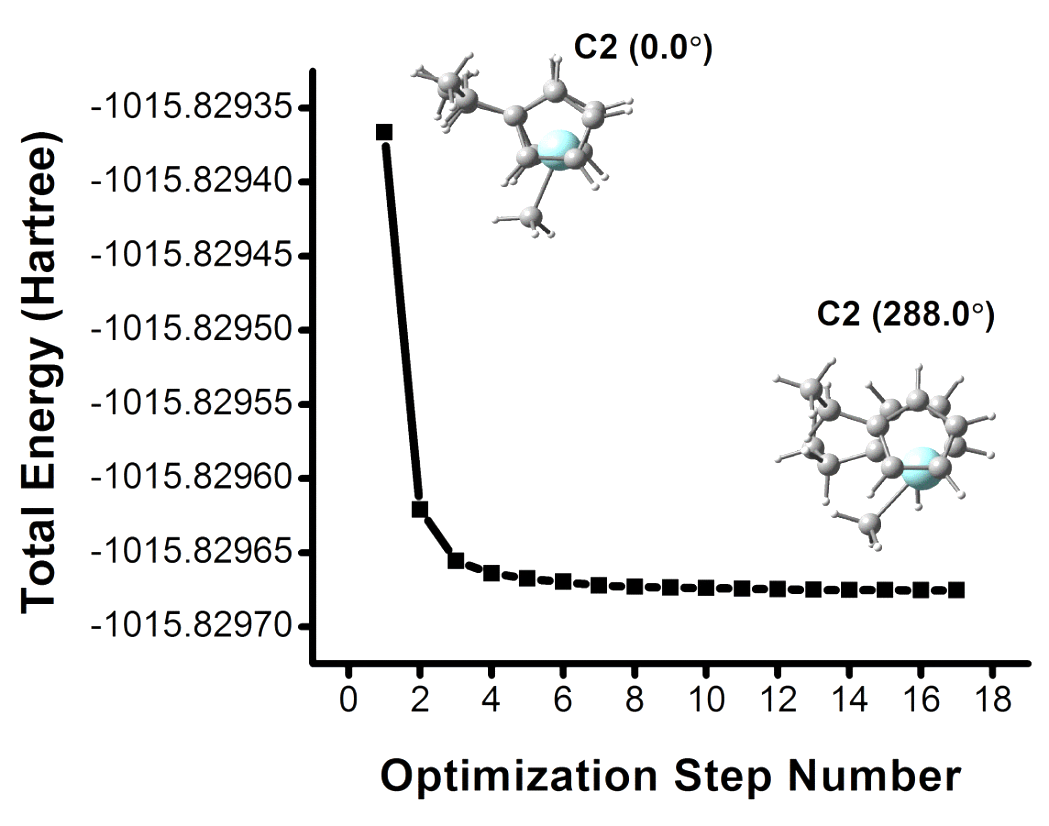


Figure S12. Results from geometry optimization starting with C2 (0.0°)/C2[1,1’] into

C2 (288.0°)/C2[1,5’] at Cation.


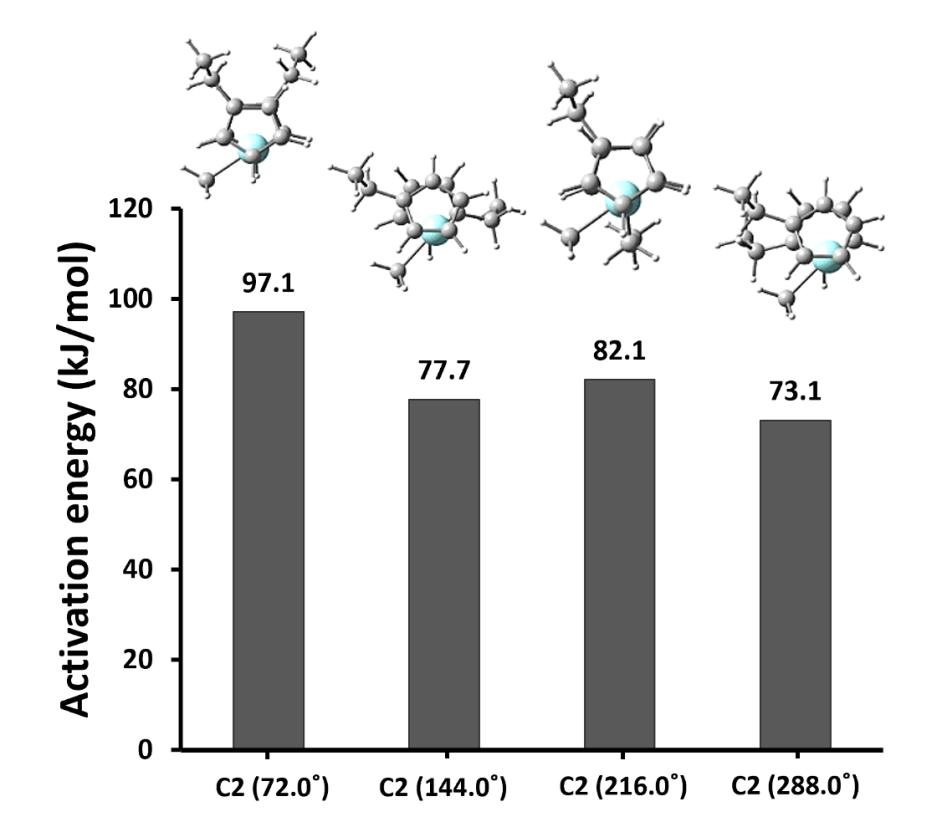


Figure S13. Activation energies (kJ/mol) of the first propagation step for C2 in different conformers.

*
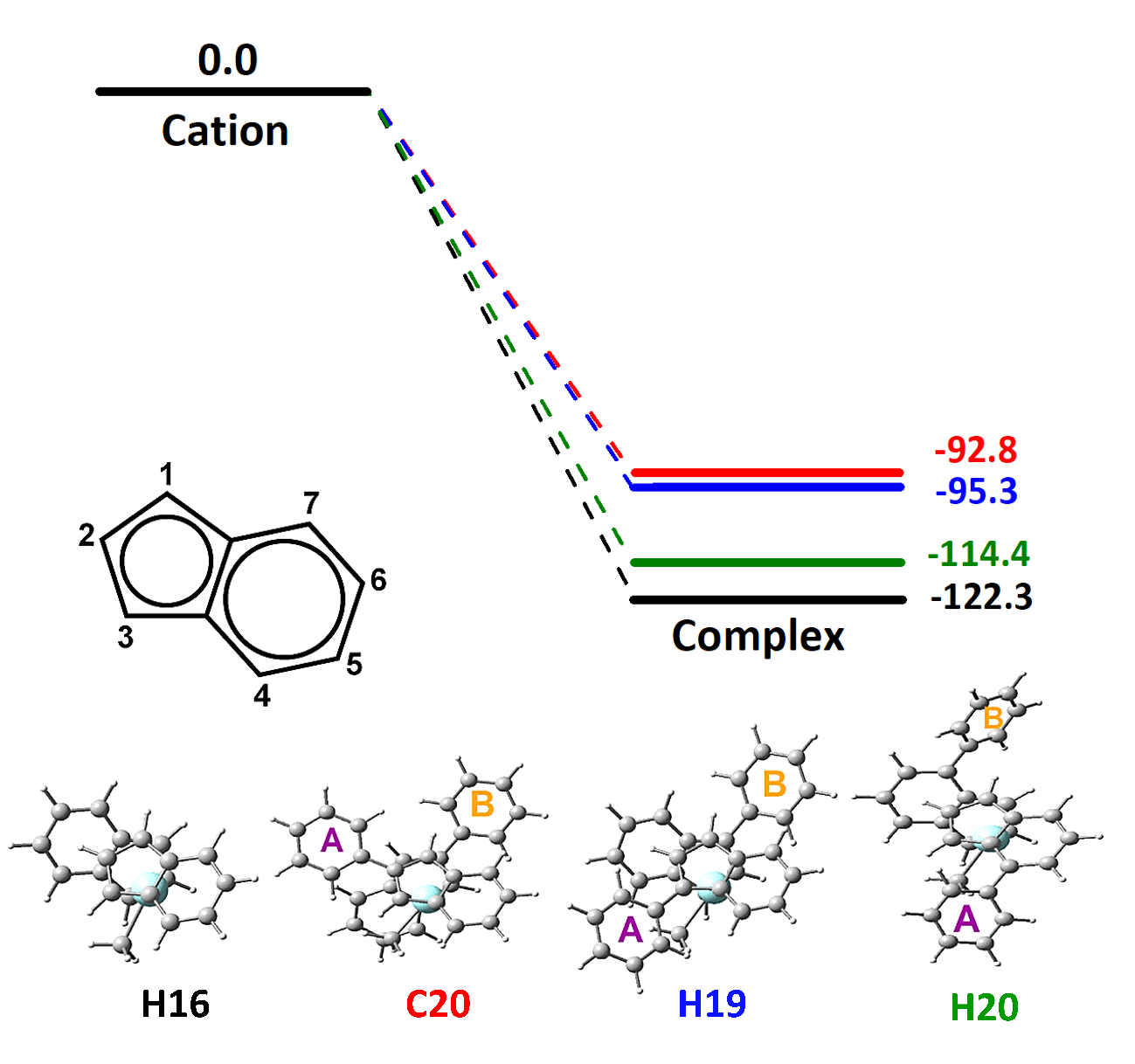
*

Figure S14. Relative energies (kJ/mol) of the initiation step for H16, C20, H19, and H20.

*
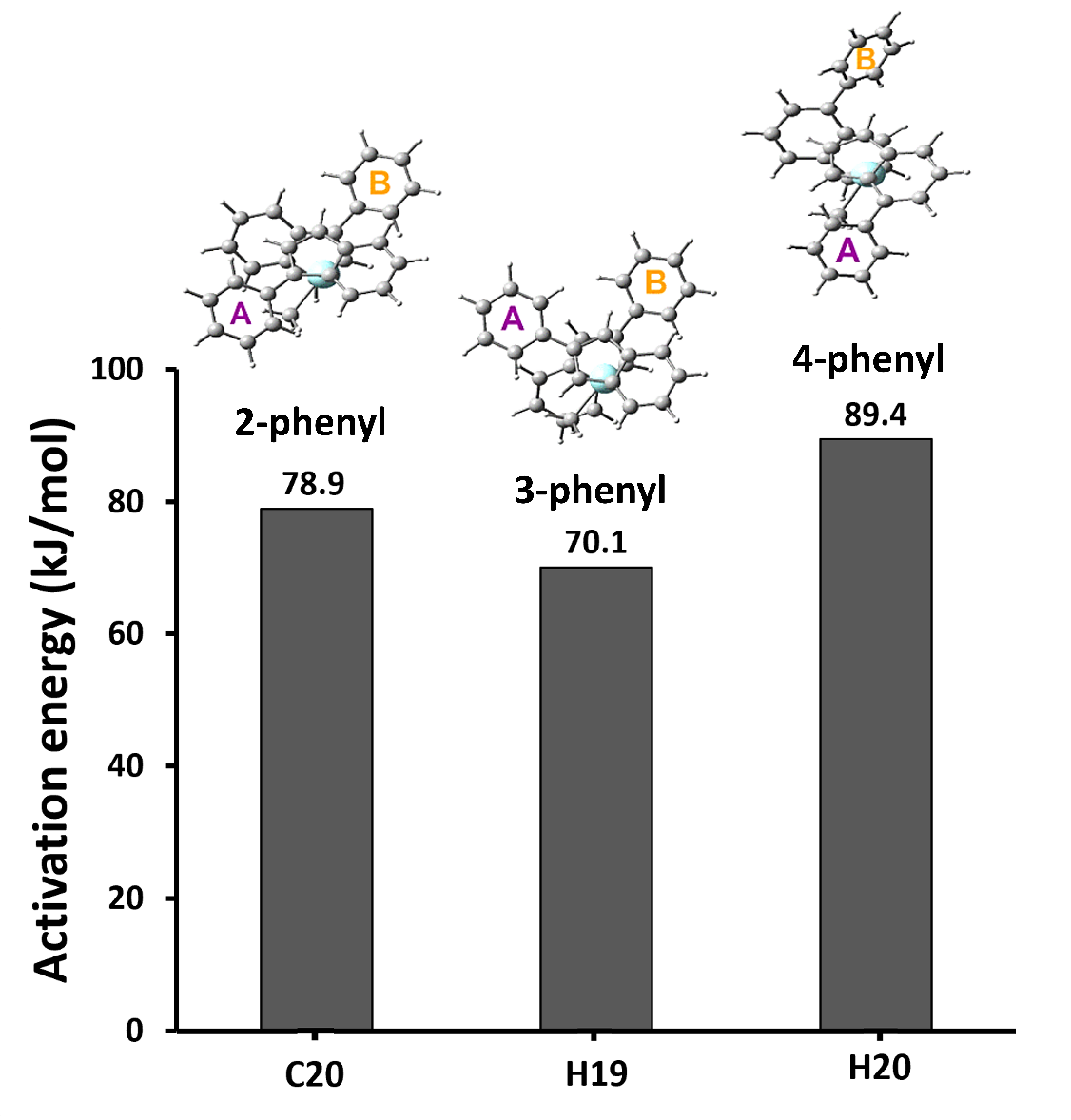
*

Figure S15. Activation energies (kJ/mol) of the first propagation step for H16, C20, H19, and H20.

*
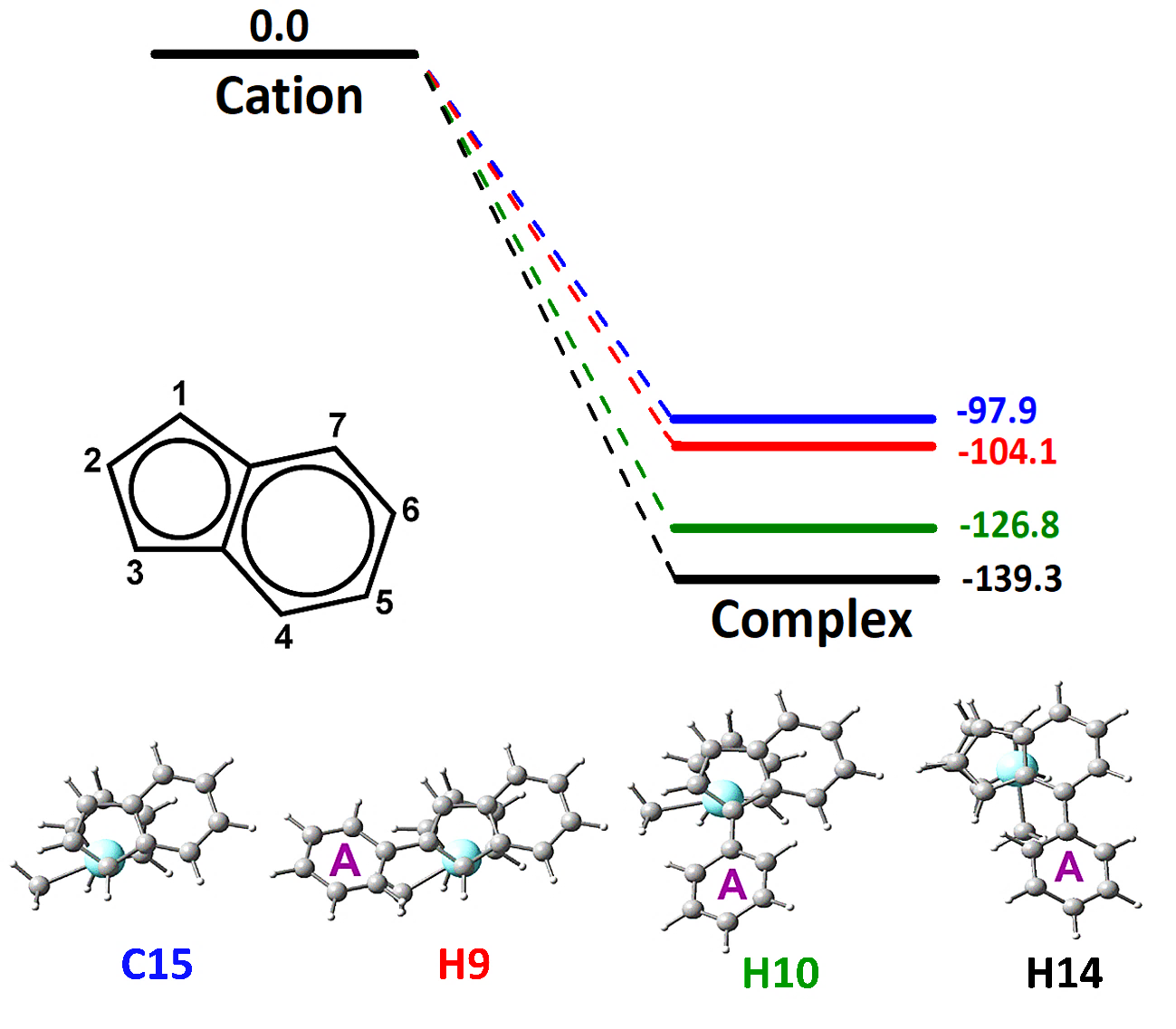
*

Figure S16. Relative energies (kJ/mol) of the initiation step for C15, H9, H10, and H14.

*
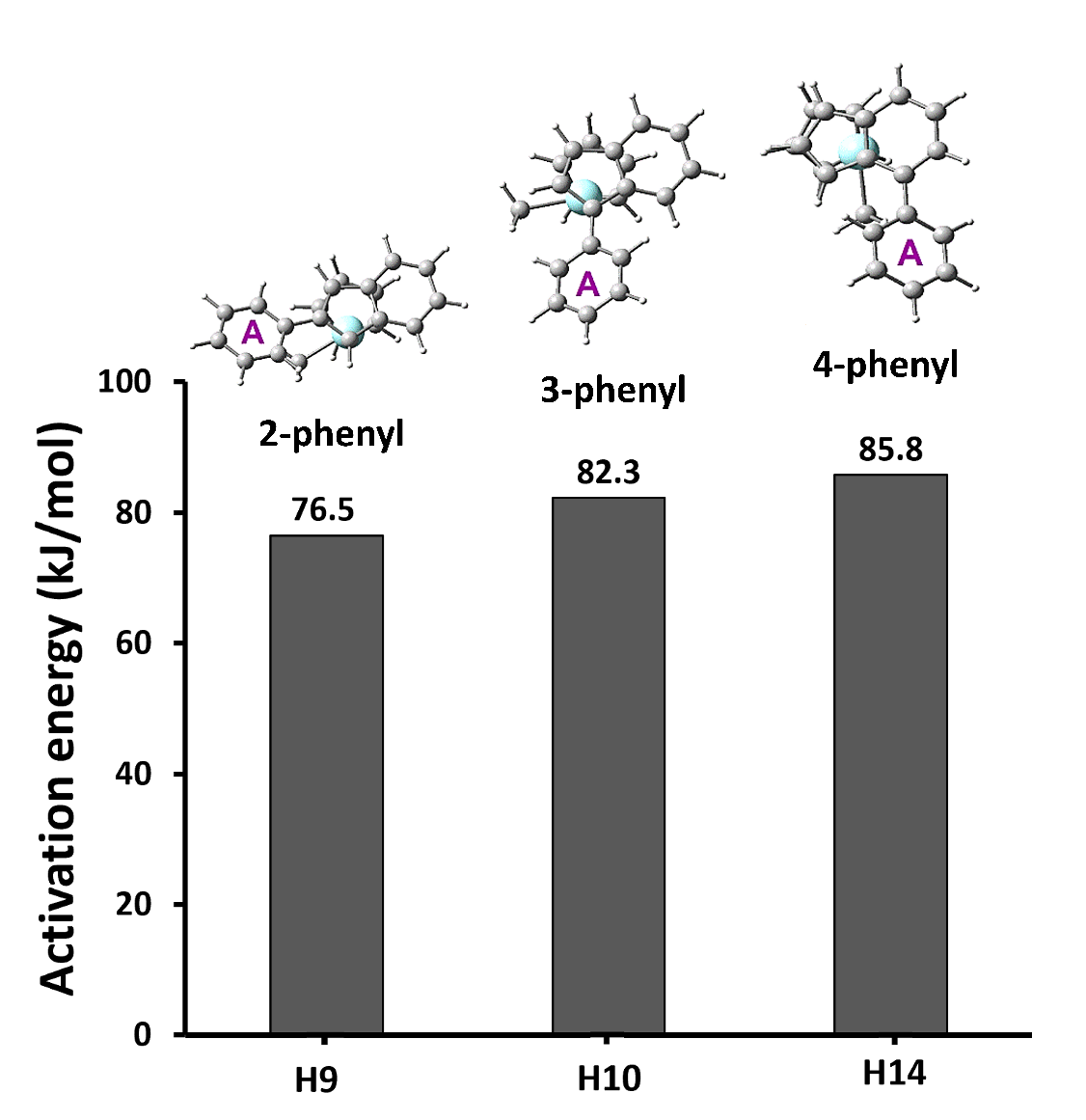
*

Figure S17. Activation energies (kJ/mol) of the first propagation step for C15, H9, H10, and H14.


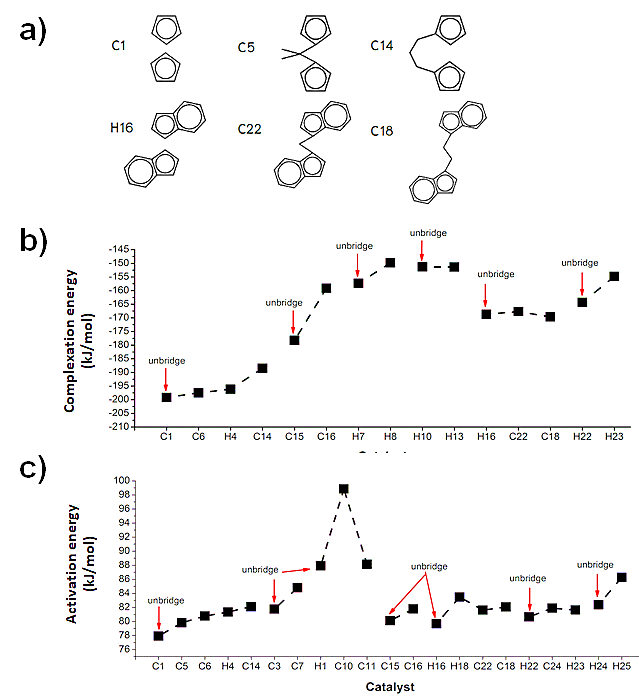


**Figure S18.** (a) Selected ligands for comparison in the bridge effect study. Profiles of complexation (b) and activation energies (c) used in the effect of bridge. Red arrows indicate the unbridged ligand as reference. (c) Selected ligands for comparison in the bridge effect study.


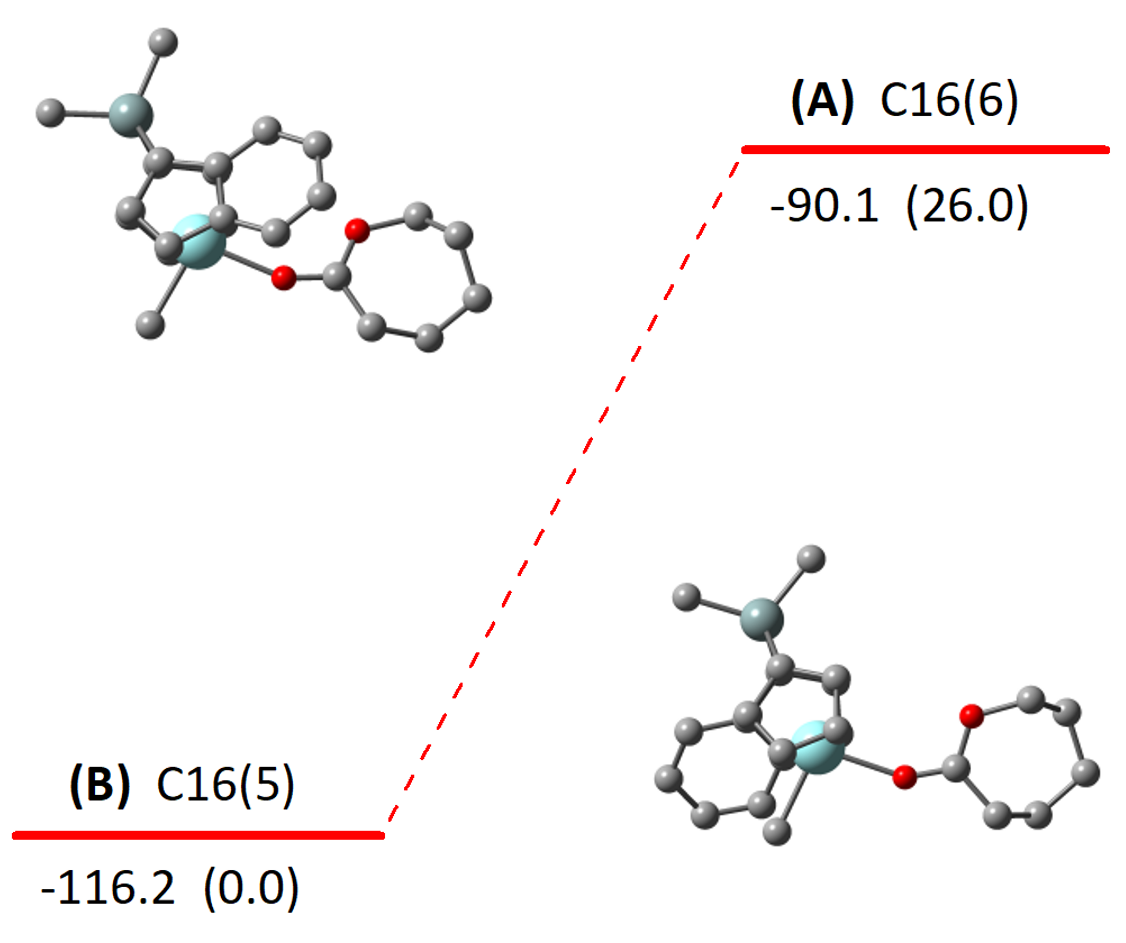


**Figure S19.** Relative energies between **C16(5)** and **C16(6).**

**VII. References**

1 Kaluzynski, K. *et al.* Cationic polymerization of cyclic trimethylene carbonate induced with initiator and catalyst in one molecule: Polymer structure, kinetics and DFT. *J. Catalysis* **415**, 200-217 (2022).

2 Nifant'ev, I. & Ivchenko, P. Coordination Ring-Opening Polymerization of Cyclic Esters: A Critical Overview of DFT Modeling and Visualization of the Reaction Mechanisms. *Molecules* **24**, 4117 (2019).

3 Nifant'ev, I. & Ivchenko, P. DFT modeling of organocatalytic ring-opening polymerization of cyclic esters: a crucial role of proton exchange and hydrogen bonding. *Polymers (Basel)* **11**, 2078 (2019).

4 Jitonnom, J., Molloy, R., Punyodom, W. & Meelua, W. Theoretical studies on aluminum trialkoxide-initiated lactone ring-opening polymerizations: Roles of alkoxide substituent and monomer ring structure. *Comp. Theor. Chem.* **1097**, 25-32 (2016).

5 Jitonnom, J. & Meelua, W. Effect of ligand structure in the trimethylene carbonate polymerization by cationic zirconocene catalysts: A “naked model” DFT study. *J. Organomet. Chem.* **841**, 48-56 (2017).

6 Hayakawa, M., Mitani, M., Yamada, T. & Mukaiyama, T. Living ring-opening polymerization of lactones using cationic zirconocene complex catalysts. *Macromol. Chem. Phys.* **198**, 1305-1317 (1997).

7 Kostakis, K., Mourmouris, S., Karanikolopoulos, G., Pitsikalis, M. & Hadjichristidis, N. Ring-opening polymerization of lactones using zirconocene catalytic systems: Block copolymerization with methyl methacrylate. *J. Polym. Sci. A Polym. Chem.* **45**, 3524-3537 (2007).
